# A minimalist self-assembly nanosystem for cancer immunotherapy via multiple immune activation

**DOI:** 10.1186/s12951-025-03464-1

**Published:** 2025-06-02

**Authors:** Weizhe Xu, Shiyuan Wang, Jiayi Zhang, Fang Wang, Zhaogang Sun, Bei Liu, Jun Ye, Hongqian Chu

**Affiliations:** 1 Translational Medicine Center, Beijing Chest Hospital, Beijing Tuberculosis and Thoracic Tumor Research Institute, Capital Medical University, Beijing, 101149 China; 2 State Key Laboratory of Bioactive Substance and Function of Natural Medicines, Institute of Materia Medica, Chinese Academy of Medical Sciences & Peking Union Medical College, Beijing, 100050 China; 3 Translational Medicine Center, Beijing Chest Hospital, Capital Medical University, Beijing, 101149 China; 4 College of Science, Minzu University of China, Beijing, 100081 China

**Keywords:** Photothermal therapy, Photodynamic therapy, Chemodynamic therapy, Immunotherapy, Self-assembly

## Abstract

In recent years, anti-tumor immunity has emerged as a central focus in cancer research, with the rapid advancement of immunotherapy heralding a new era in cancer treatment. Despite the significant potential of immunotherapy, the use of single-agent approaches or limited combination therapies has not consistently yielded optimal therapeutic outcomes. The strategic and controlled integration of diverse immune activation techniques within a single nanoparticle, utilizing a straightforward and universal methodology, continues to present a substantial challenge. Self-assembly, as a simple synthesis method, offers the possibility of combining multiple therapeutic approaches through straightforward means. In this study, we developed a novel approach to construct a biocompatible nanosystem, named Cu-ICG-CpG-FA (CICF), which was synthesized through one-pot coordination-driven self-assembly of Cu^2+^ ions, CpG oligonucleotides and indocyanine green (ICG), followed by a surface modification with folic acid. Folic acid, as a ligand, can bind to folic acid receptors expressed on the surface of tumor cells. Cu^2+^ facilitates chemodynamic therapy (CDT) through the Fenton reaction. ICG serves as a therapeutic for photothermal therapy (PTT) and photodynamic therapy (PDT). Moreover, CDT and PTT/PDT can induce immunogenic cell death (ICD), which is further enhanced by the immune-stimulating effect of CpG, thereby improving the tumor immunosuppressive microenvironment. Therefore, CICF provides a simple and efficient approach to synergistic cancer immunotherapy with promising clinical applications.

**Graphical Abstract:**

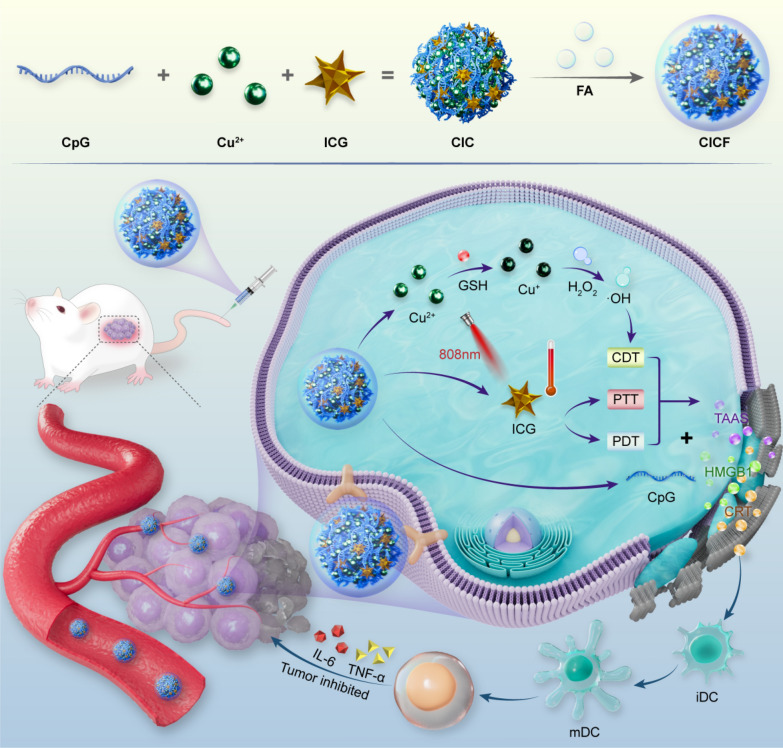

**Supplementary Information:**

The online version contains supplementary material available at 10.1186/s12951-025-03464-1.

## Introduction

Cancer treatment remains a significant global public health challenge [1]. Conventional therapies often suffer from limitations such as low selectivity and severe side effects. Immunotherapy is becoming an important treatment modality for cancer. However, the immunosuppressive characteristics of the tumor microenvironment (TME) severely limit the efficacy of clinical immunotherapy [2]. Single-agent immunotherapy often exhibits low response rates and poor outcomes, making efficient multi-pathway immune activation significant for cancer immunotherapy [3]. In recent years, as the number of studies on combination immunotherapy increased, most trials have compared results with standard chemotherapy, yet the outcomes remain unsatisfactory [4]. There is an urgent need to establish optimal combination strategies to achieve effective anti-tumor immunity.

Recently, nanomaterials, which enable delicate structure tailoring and good biocompatibility, have shown great potential for integrating multiple functionalities [5, 6]. To improve efficacy and safety, numerous novel tumor-therapeutic strategies have emerged, including photothermal therapy (PTT) [7, 8], photodynamic therapy (PDT) [9, 10], chemodynamic therapy (CDT) [11, 12], sonodynamic therapy (SDT) [13, 14]. In addition to relying on kinetic mechanisms to kill tumor cells, the aforementioned treatment modalities can also induce the release of tumor-associated antigens (TAA) [15]. Therefore, when used in combination with immunoadjuvants, they can enhance anti-tumor immune responses. Despite exhaustive efforts, current approaches are often hampered by complex materials synthesis and formulation. A primary challenge in the domain of multifunctional nanomaterials is the simplification of the synthesis process, alongside improvements in scalability and functionality.

Coordination-driven self-assembly is an effective supramolecular strategy for constructing functional molecular architectures and materials [16, 17], encompassing a variety of structures including coordination polymers [18], metal cages [19], and supramolecular polymer gels [20]. The integration of organic and inorganic components at the molecular level enhances structural complexity, enabling more advanced functionalities and a broader range of applications [21, 22]. Particularly, nanoscale coordination polymers have become a research focus due to their widespread applications in adsorption, selective catalysis, optical materials, and nanomedicine. [23, 24] Current synthesis methods commonly involve the coordination self-assembly of metal ions with small organic molecules (such as 2-methylimidazole, porphyrins) [25, 26] or biomolecules (such as tannic acid, dipeptides) [27, 28]. Researchers have already utilized metals (such as Cu and Fe) to assemble nanomaterials for amplifying immune responses and enhancing anti-tumor efficiency [29–31]. However, to our knowledge, the controlled synthesis of a minimalist coordination-driven self-assembly multi-modal therapeutic nanosystem for amplified cancer immunotherapy has yet to be reported.

**Scheme 1:**
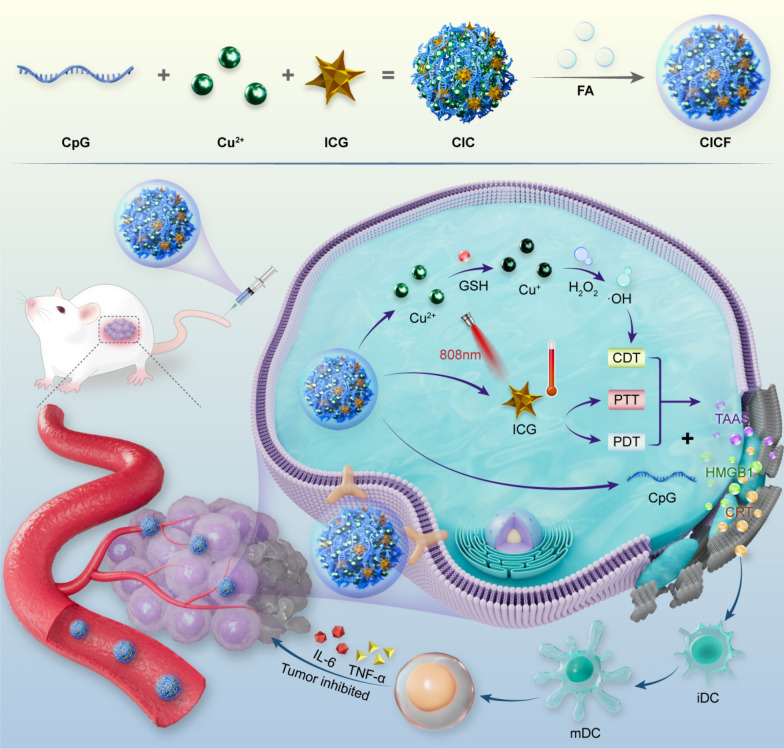
A schematic representation of the synthesis of CICF and its application in tumor treatment through the integration of photodynamic therapy (PDT), photothermal therapy (PTT), chemodynamic therapy (CDT), and immunotherapy

Herein, we report a simple and facile strategy to construct a coordination-driven self-assembly, named CICF, for efficient combination therapy (Scheme 1). With the aid of Cu^2+^ ions, CpG oligonucleotides and indocyanine green (ICG) could be co-assembled into a uniform nanosphere. Subsequent addition and mixing with folic acid (FA) imparted tumor-targeting capabilities, resulting in a minimalist assembled nanosystem of CICF. Following tumor-targeted delivery, the nanosystem undergoes degradation in the acidic tumor microenvironment (TME), releasing Cu^2+^ which is reduced to Cu^+^ by glutathione (GSH). Cu^+^ then reacts with endogenous hydrogen peroxide (H_2_O_2_) to generate hydroxyl radicals (•OH) to mediate CDT. Furthermore, near-infrared (NIR) irradiation triggers PTT and PDT via ICG. The combination of CDT with PTT/PDT could trigger tumor-associated antigens (TAA) release, activating antitumor immunity in synergy with CpG immune adjuvant. Together, the as-synthesized nanosystem offers a novel approach for enhanced combination cancer immunotherapy.

## Results and discussion

### Synthesis and characterization of CICF

**Fig. 1:**
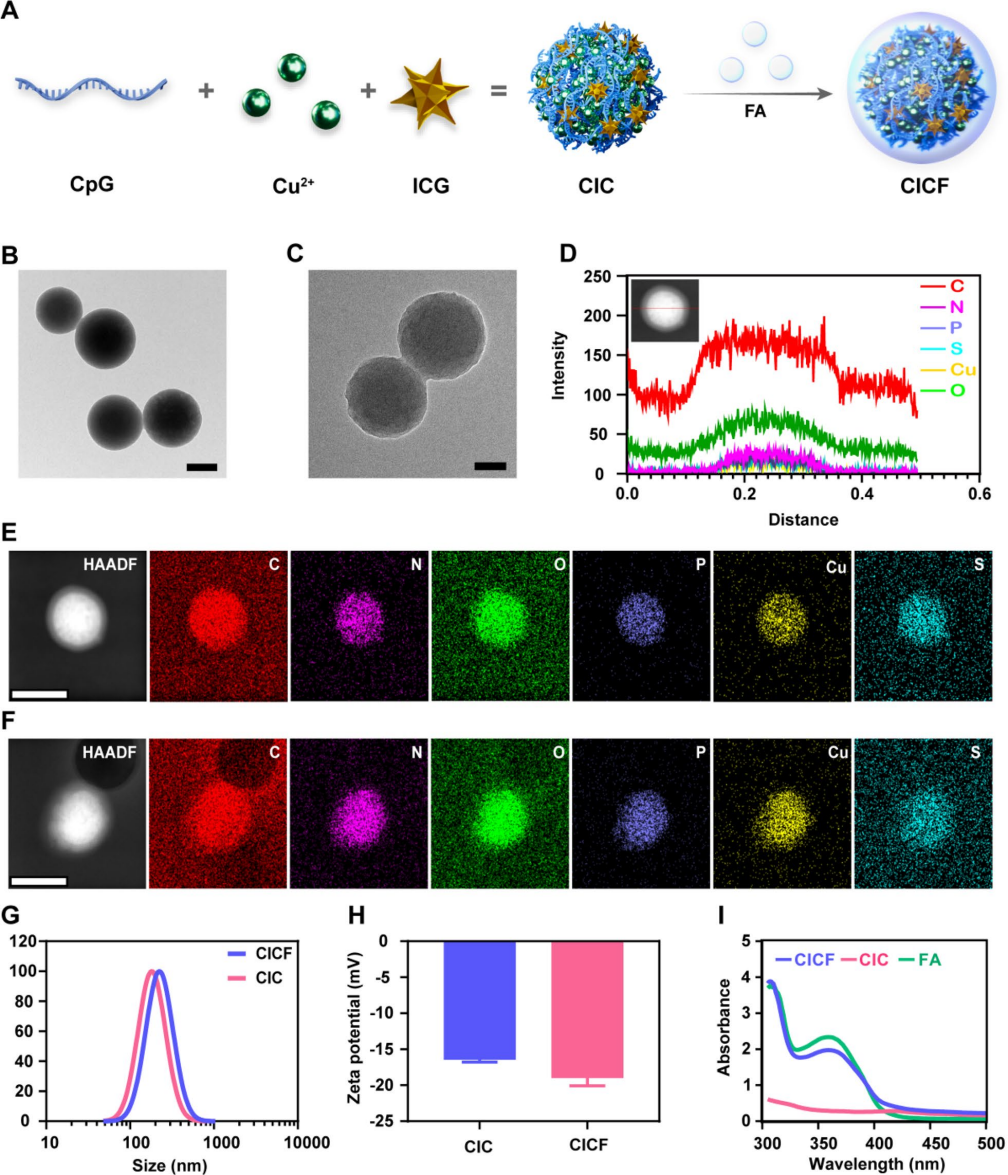
**A** The efficient coordination-driven self-assembly of cargos during the synthesis process of CICF. TEM showed the morphology of CICF at **B** low magnification (scale bar: 200 nm) and **C** high magnification (scale bar: 100 nm). **D** EDS line scanning profiles of CICF. TEM element mapping images of C, N, O, P, S, and Cu in **E** CICF and **F** CIC. Scale bar: 200 nm. **G** DLS analysis of the hydrodynamic size of CIC and CICF. **H** Zeta potential of CIC and CICF. **I** UV–vis absorption spectra of FA, CIC, and CICF. Results are presented as means ± SD (n = 3)

CpG oligonucleotide is a type of immune adjuvant that can activate Toll-like receptor 9 (TLR9) to trigger an immune response and enhance anti-tumor immune responses [32]. ICG is commonly used as a fluorescent dye in the medical field and can trigger its powerful photothermal and photodynamic effect upon irradiation with an NIR laser [33]. Previous studies reported that metal ions, DNA, and small molecule drugs can self-assemble into nanoparticles through coordination [28, 34]. In this study, with the aid of Cu^2+^ ions, CpG and ICG molecules were co-assembled into a uniform nanosphere, named Cu-ICG-CpG (CIC) (Fig. 1A). The element distribution of CIC was analyzed through the high-angle annular dark-field scanning transmission electron microscopy-energy-dispersive X-ray spectroscopy (HAADF-STEM-EDS) elemental mapping (Fig. 1F). P (derived from CpG), S (derived from ICG), and Cu elements were homogeneously distributed in the CIC nanoparticles, demonstrating the successful self-assembly of CpG, ICG, and Cu^2+^ ions.

As a vitamin, folic acid (FA) has become an important ligand in targeted cancer therapy because it can bind to a tumor-associated antigen called the folate receptor (FR). Here, FA was modified on the surface of CIC nanoparticles through coordination. After modification with FA, CICF binds specifically to folate receptors on the surface of tumor cells through a receptor-ligand interaction mechanism. This enables more effective recognition, binding, and entry into folate receptor-positive tumor cells, thereby enhancing targeting capability [35]. Representative transmission electron microscope (TEM) images of CICF (Fig. 1B, C). Monodisperse nanoparticles with a diameter of about 200 nm can be clearly observed. Scanning electron microscope (SEM) showed a similar morphology of CICF compared with CIC nanoparticles (Figure S1). Dynamic light scattering (DLS) results indicated the average diameter of CICF increased from 201.96 ± 1.95 nm to 216.10 ± 4.8 nm (Fig. 1G). The zeta potential of CICF decreased from −16 mV to −19 mV (Fig. 1H) due to the existence of carboxyl negative groups in FA. Furthermore, we observed characteristic peaks of FA in CICF (Fig. 1I), directly demonstrating the successful modification of FA. Therefore, there are certain differences in properties between CIC and CICF. The HAADF-STEM-EDS elemental mapping of CICF also illustrated a uniform distribution of P (derived from CpG), S (derived from ICG), and Cu elements (Fig. 1E). These homogeneously distributed elements within a single CICF nanoparticle are also confirmed by the EDS line scan profiles (Fig. 1D). Then the quantitative analysis of ICG molecules contained in the CICF nanoparticles was conducted. An absorbance-concentration plot was tested based on a series of ICG concentrations, resulting in a fitted straight line y = 0.04015x + 0.01111, R^2^ = 0.998 (Figure S2). According to the linear relationship of ICG, the loading content of ICG in CICF nanoparticles was 64%. Similarly, the loading content of CpG was calculated as 35% (Figure S3). The high loading efficiency of ICG and CpG in CICF was based on the efficient coordination-driven self-assembly of cargos during the synthesis process of CICF. In addition, to ascertain the acid-degradation capability of CICF, we conducted a comparative experiment utilizing solutions with pH values of 6.4 and 7.4 to monitor the release of ICG from CICF (Figure S6). Under acidic conditions (pH 6.4), there was a marked increase in the release of ICG. These findings demonstrate that CICF possesses robust acid-degradation properties, enabling the effective release of ICG in an acidic environment.

**Fig. 2:**
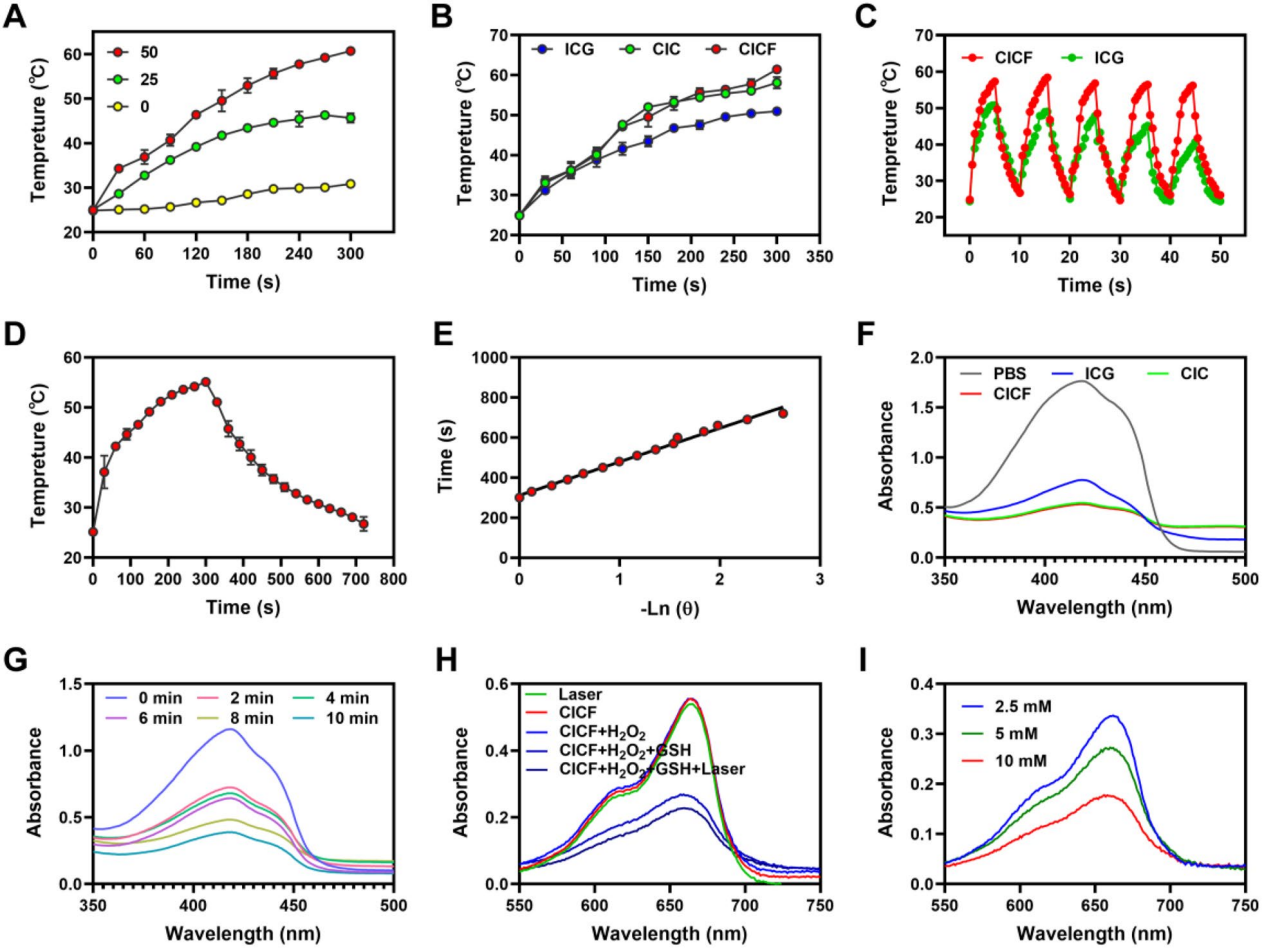
**A** Temperature curves with CICF at different concentrations under NIR laser irradiation (1.0 W/cm^2^). **B** Temperature curves of different solutions with the same amount of ICG upon the NIR laser irradiation (1.0 W/cm^2^). **C** Temperature curves of ICG and CICF (100 µg/mL) over five laser irradiation ON/OFF cycles. **D** Photothermal effect CICF with NIR laser irradiation (1.0 W/cm^2^). **E** Linear time data versus − ln(θ) obtained from the cooling period of (Fig. 2D). **F** DPBF was incubated with different groups and the UV–vis absorption spectra after NIR laser irradiation were compared to the generation of ^1^O_2_. **G** UV–vis absorption spectra of DPBF after incubation with CICF at different times following NIR laser irradiation for comparison of ^1^O_2_ generation. **H** UV–vis absorption spectra of MB aqueous solution under different treatment conditions. **I** UV–vis spectra of MB aqueous solution treated with CICF and GSH at different concentrations for 30 min. Results are presented as means ± SD (n = 3)

Next, we evaluated the photothermal ability of CICF under NIR laser irradiation. Under the irradiation of the NIR laser, the temperature of the CICF solution (50 μg/mL) rises rapidly, reaching the highest temperature of 61 °C at about 5 min light irradiation (1.0 W/cm^2^) (Fig. 2A). An increase in the concentration of CICF will result in the generation of more heat when exposed to near-infrared laser irradiation. Notably, the aqueous solutions containing free ICG, CIC, and CICF presented similar temperature profiles, indicating that the self-assembly of nanoparticles and the FA modification cannot impact the photothermal effect of ICG molecules (Fig. 2B). The temperature increase curves of different concentrations of CICF showed a concentration-dependent photothermal behavior (Fig. 2A). To determine the photothermal conversion efficiency of CICF, the heating and cooling curve of the aqueous solution of CICF was tested (Fig. 2D). As presented in Fig. 2D, E, the photothermal conversion efficiency was calculated to be 33.35%. In addition, CICF still showed a high photothermal conversion effect after 5 ON/OFF irradiation cycles (1 W/cm^2^), which proved that CICF had excellent photothermal stability (Fig. 2C). The photothermal effect of CICF is similar to previously reported results of materials constructed with ICG [36, 37]. Both the high photothermal conversion efficiency and the good photothermal stability suggest that CICF holds great promise for tumor photothermal therapy.

Methylene blue (MB) can be degraded by·OH and is considered as an indicator for Fenton reaction [11]. Herein, we chose MB as an indicator to evaluate the ability of CICF to produce·OH via a Fenten-like reaction. After incubation of CICF with H_2_O_2_ and GSH (CICF + H_2_O_2_ + GSH) for 30 min in MB solution, the absorbance of MB significantly decreased (Figs. 2H and S4). By contrast, the absorption intensity of MB remained unchanged after CICF, laser, or CICF + H_2_O_2_ treatments. These results indicate that GSH reduces Cu^2+^ to Cu^+^. Subsequently, the reduced Cu + reacts with H_2_O_2_, catalyzing the decomposition of H_2_O_2_ and generating hydroxyl radicals (·OH) with strong oxidizing properties. Moreover, the decrease in absorbance of MB in the CICF solution exhibited a concentration-dependent catalytic behavior (Fig. 2I). To verify that CICF could effectively convert GSH to GSSG, the GSH indicator of 5,5'-dithiobenzene 2-(nitrobenzoic acid) (DTNB) was used to test the GSH consumption during the ·OH generation. The colorless DTNB can be reacted with GSH to generate a yellow compound of TNB, therefore providing a means for assessment of GSH consumption [38]. GSH levels decreased with increasing concentrations of CICF, indicating the effective reduction of GSH induced by CICF (Figure S5).

ICG can be used not only as a photothermal agent but also as a photosensitizer for photodynamic therapy (PDT). To study the ability of NIR to produce singlet oxygen (^1^O_2_), the production efficiency of ^1^O_2_ was evaluated by using 1, 3-diphenylisobenzofuran (DPBF). DPBF generally has a characteristic absorption peak near 420 nm, and its intensity decays irreversibly in the presence of ^1^O_2_ [9]. CICF exhibits a similar reduction in absorbance values at NIR laser compared to free ICG, indicating effective ^1^O_2_ production (Fig. 2F). In addition, after NIR laser irradiation of CICF, the absorbance value of DPBF significantly decreased with the increase of time (Fig. 2G).

### Cellular internalization and ^1^O_2_ generation of CICF

**Fig. 3:**
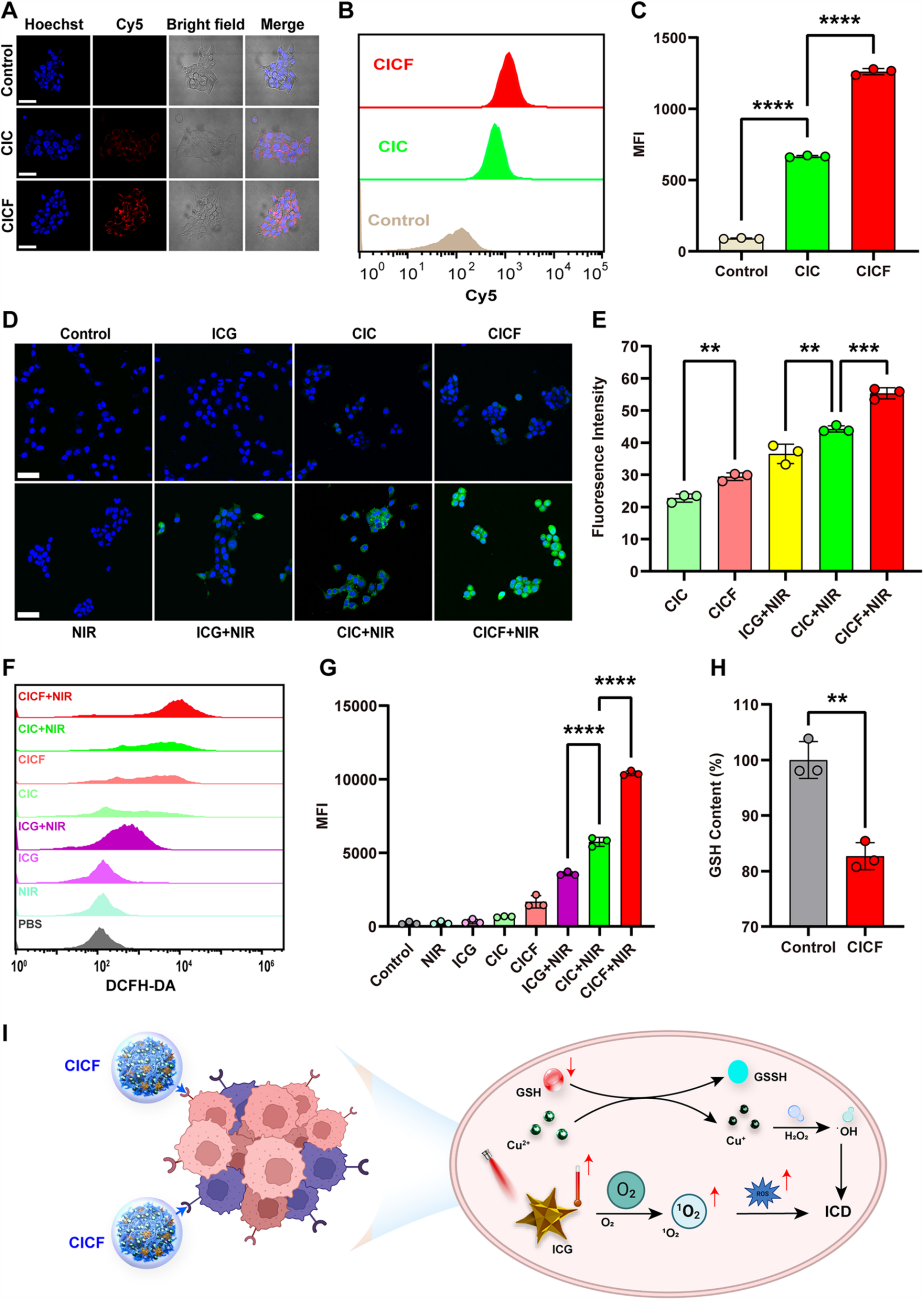
**A** CLSM images of 4 T1 cells incubated with CIC-Cy5 or CICF-Cy5 for 4 h. Scale bar: 50 μm. **B** Flow cytometric analysis of the cellular uptake of 4 T1 cells treated with CIC-Cy5 or CICF-Cy5 for 4 h and **C** the corresponding normalized mean fluorescence intensity (MFI) of (**B**). **D** CLSM images of ROS in 4 T1 cells treated with or without NIR by different components of drugs. Scale bar: 50 μm. **E** The corresponding quantifications of ROS (**D**). **F** Flow cytometric analysis of ROS accumulation after treatment with CIC or CICF and **G** the corresponding normalized mean fluorescence intensity (MFI). **H** Intracellular relative GSH levels after CICF incubation. Results are presented as means ± SD (n = 3). The difference was statistically significant: **p < 0.01, ***p < 0.001, ****p < 0.0001

Encouraged by the effectiveness of PTT, PDT, and CDT, we further validated the effectiveness of CICF in vitro. Efficient internalization of materials by tumor cells is a prerequisite for achieving therapeutic effects. CICF specifically binds to folate receptors on the surface of tumor cells, thereby more effectively recognizing, binding to, and entering folate receptor-positive 4 T1 cells (Figs S7–S10). So, we evaluated the intracellular uptake efficiency of CIC and CICF by 4 T1 cells (expressed FA receptor) using confocal laser scanning microscopy (CLSM) and flow cytometry. The cells treated with CICF (CpG was labeled with Cy5) showed more red fluorescence signals than those incubated with CIC (Fig. 3A), validating that FA ligands can effectively drive the accumulation of CICF into tumor cells. In addition, flow cytometry analysis (Fig. 3B, C) showed that the fluorescence intensity of 4 T1 cells incubated with CICF was 2.4-fold higher than that of cells treated with CIC. We further investigated the subcellular localization of CpG internalization. Lysosomes were labeled with LysoTracker Green. As shown in Figure S11, the CpG in CICF was delivered to the lysosomes of the cells, and a portion of CICF escaped from the lysosomes, which may contribute to the functional efficacy of CICF.

Subsequently, we identified the mechanism of killing tumor cells (Fig. 3I). However, the therapeutic efficiency of CDT often suffers from high concentrations of glutathione (GSH). Cu^2+^ can be reduced to Cu^+^ by depleting overexpressed GSH, thereby enhancing CDT [39]. To verify that CICF can effectively reduce the concentration of GSH, a GSH assay kit was utilized to test the intracellular GSH consumption. The intracellular GSH content was reduced to ~ 80% after 12 h treatment with CICF, indicating effective intracellular GSH consumption (Fig. 3H). Then the intracellular ·OH generation by CICF was investigated by using dichlorofluorescein diacetate (DCFH-DA) as ·OH indicator. CIC and CICF produce only weak green fluorescence signals under non-laser conditions. The 4 T1 cells incubated with ICG, CIC, and CICF following laser irradiation all showed significant green fluorescence compared to without irradiation. CICF-treated cells showed the strongest green fluorescence, indicating that targeted CICF produced more reactive oxygen species (ROS) (Fig. 3D, E). The production of ROS was further verified by flow cytometry. The production of ROS induced by CICF + NIR was significantly higher than that of other groups, and the flow cytometry results were consistent with the CLSM experimental results (Fig. 3F, G). Therefore, CICF could produce more ROS under NIR laser irradiation.

### Antitumor performance of CICF in vitro

**Fig. 4:**
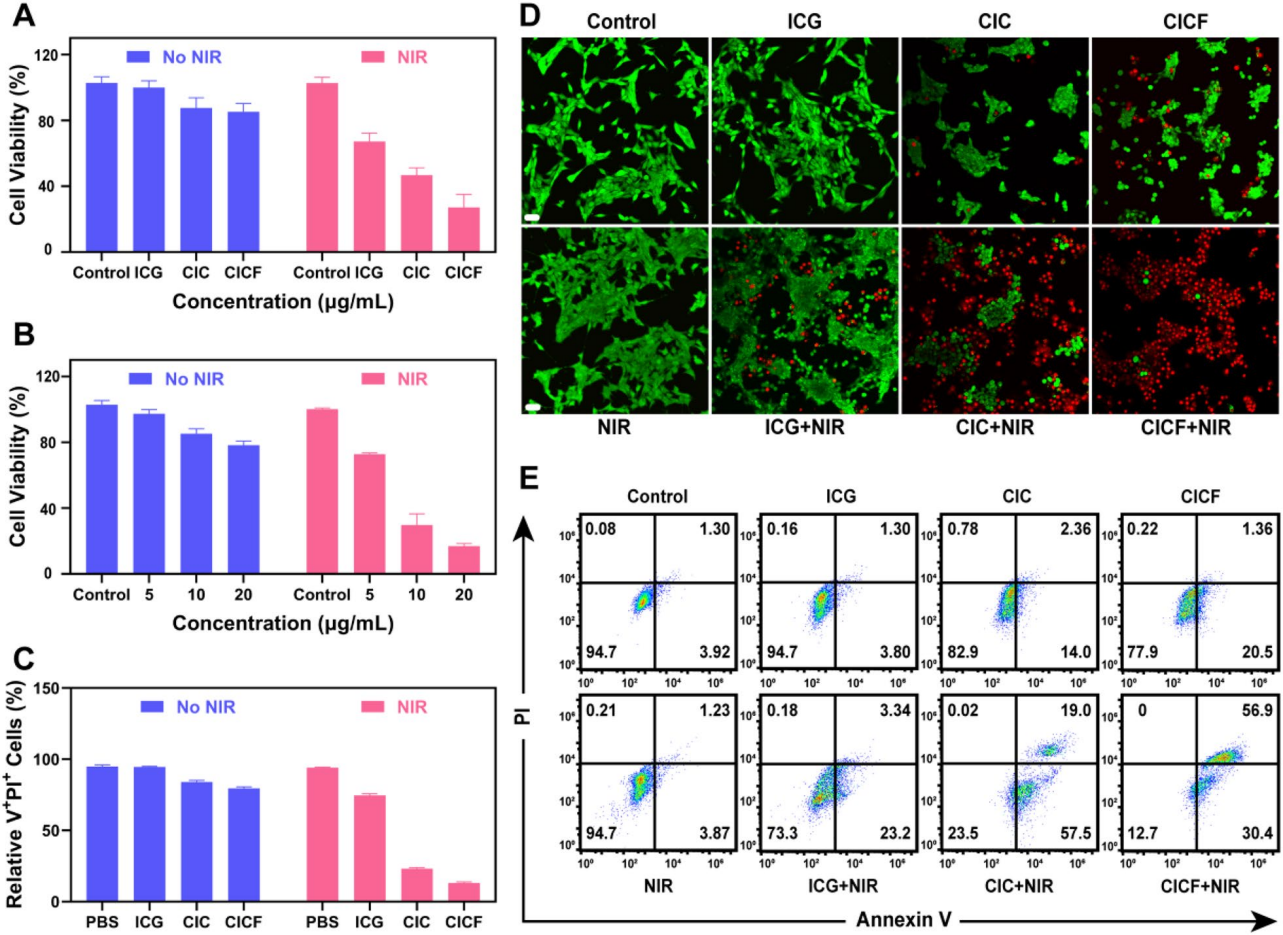
**A** Cell viability of 4 T1 cells treated with ICG, CIC, and CICF under laser or no laser conditions. **B** Cell viability of 4 T1 cells treated with different concentrations of CICF (from 5 to 20 μg/mL) under laser or no laser conditions. **C** Quantification of PI +/Annexin V-FITC + 4 T1 cells after the indicated treatment. **D** CLSM images of 4 T1 cells under different treatments were stained with Calcein-AM (green, live cells) and PI (red, dead cells). Scale bar: 50 μm. **E** Apoptosis of 4 T1 cells stained with Annexin V-FITC/PI was analyzed by flow cytometry. Results are presented as means ± SD (n = 3)

The in vitro cytotoxicity of 4 T1 cells irradiated at a power of 2 W/cm^2^ for 5 min was evaluated using CCK-8 assay (Figure S12). At this power, not only did each treatment group almost completely kill all tumor cells, but the control group also showed significant cell damage. Therefore, while maintaining the drug concentration in each treatment group, we adjusted the irradiation power to 1 W/cm^2^, with the irradiation time remaining at 5 min, to re-evaluate the anti-tumor efficacy of each group. As shown in Fig. 4A, control or free ICG treatments did not show an obvious cell-killing effect, demonstrating the negligible toxicity of the NIR light irradiation or free ICG to 4 T1 cells. The cells treated with CIC or CICF without NIR induced a low cytotoxicity because of the generation of ·OH. In contrast, the cells treated with CIC + NIR or CICF + NIR showed significant cytotoxicity compared with other groups, demonstrating that combined therapy could effectively improve the therapeutic effect. Notably, NIR-irradiated CICF showed higher cytotoxicity compared to that of the cells treated with CIC + NIR, indicating an enhanced antitumor efficiency of CICF through the FA targeting effect. A dose-dependent cytotoxicity of CICF upon NIR laser irradiation was also tested (Fig. 4B), verifying the excellent cell-killing effect of CICF as an anti-tumor agent. As shown in Figure S13, the survival rate of normal 16HBE cells was significantly higher than that of breast cancer 4 T1 cells at the same concentration of CICF, especially at 10 and 20 μg/mL concentrations. This finding suggests that CICF has good anti-tumor selectivity, which may be due to the fact that the modified FA on the surface of CICF can specifically bind to the overexpressed FA receptor on the surface of 4 T1 cells to promote the cellular uptake of CICF (Figs S9 and S10). The cell-killing efficiency of free ICG, CIC, and CICF against 4 T1 cells was further confirmed using live/dead cell staining of calcein acetoxymethyl ester (Calcein-AM) and propyl iodide (PI). The killing effect of CICF + NIR on 4 T1 cells is more significant than that of other treatments (Fig. 4C, E).

### CICF-induced immunogenic death

**Fig. 5:**
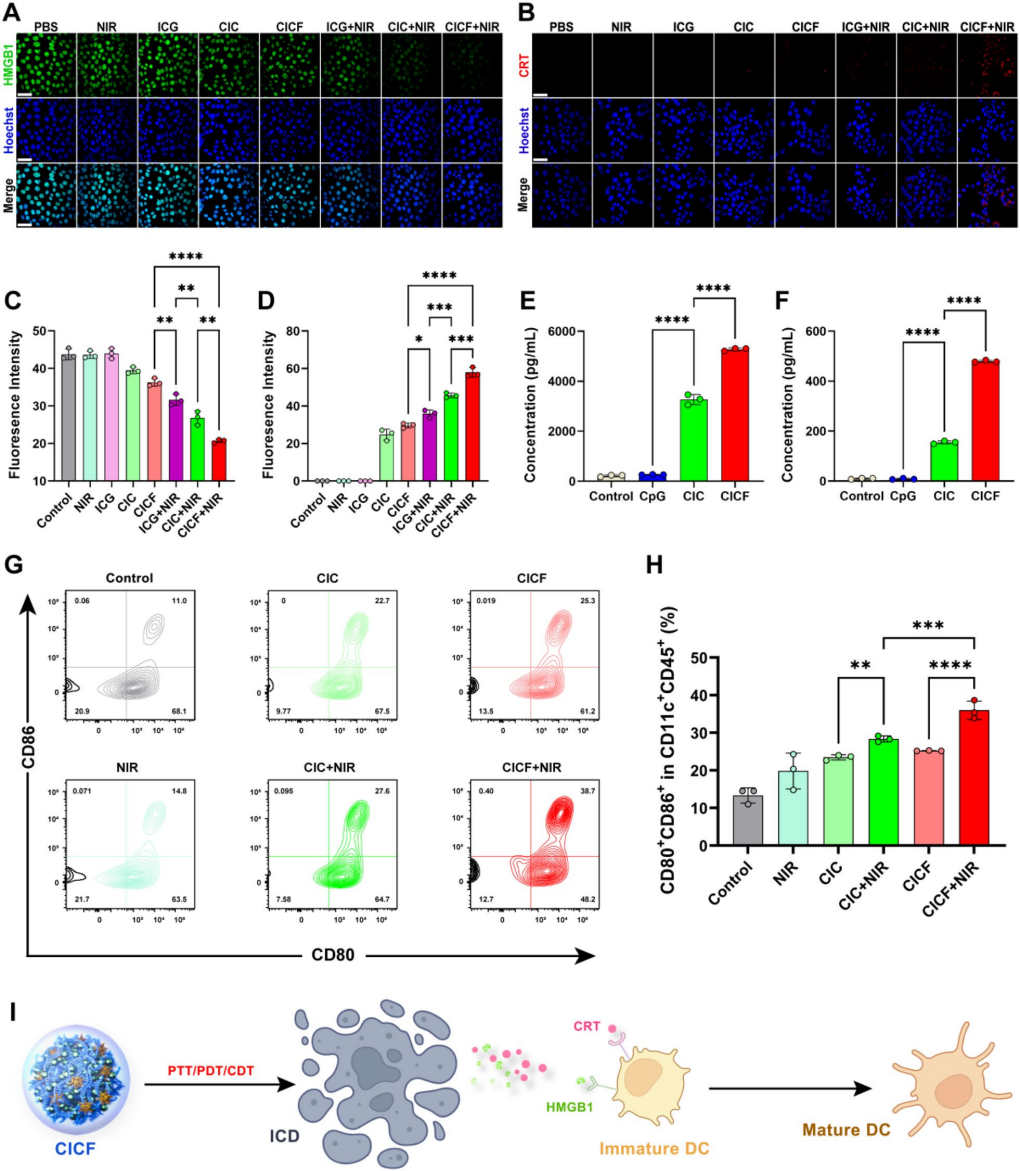
**A** Images of CLSM released by HMGB1 in 4 T1 cells after different treatments. Scale bar: 50 μm. **B** Images of CLSM expressed by CRT in 4 T1 cells after different treatments. Scale bar: 50 μm. The corresponding quantifications of CLSM images fluorescence intensity of HMGB1 (**C**) and CRT (**D**). The cytokine concentrations of **E** TNF-α and **F** IL-6 in the supernatant of RAW264.7 cells cultured under different treatment conditions were detected by ELISA. **G** Flow cytometry assay of mature DCs (CD11c + CD80 + CD86 +) in the different groups. **H** Corresponding quantitative percentages of mature DCs in different groups. **I** immunogenic cell death activates DC cell maturation. Results are presented as means ± SD (n = 3). The difference was statistically significant: *p < 0.05, **p < 0.01, ***p < 0.001, ****p < 0.0001

PTT, PDT, and CTD were able to trigger host immunity, which provided promising potential in combination with immunotherapy [40]. Considering the functions of CICF contain PTT/PDT/CDT, we verified whether CICF could induce immunogenic cell death (ICD). The occurrence of ICD is accompanied by the release of a series of ICD-related molecules, including high mobility group box 1 (HMGB1) and calreticulin (CRT) [41]. CRT translocates from the endoplasmic reticulum to the cell membrane surface, a process that facilitates antigen presentation. Simultaneously, HMGB1 is released from the nucleus to the extracellular space, acting as a damage-associated molecular pattern (DAMP), which activates immune cells (Fig. 5I). The extracellular release of HMGB1 was first detected by CLSM. During ICD, tumor cells would translocate HMGB1 from the nucleus to the cytoplasm, followed by its secretion into the extracellular milieu. As shown in Fig. 5A, the levels of HMGB1 were marginally reduced in cells treated with CIC or CICF compared to the control group. The cells treated with CIC + NIR showed a weak fluorescence intensity, indicating a more efficient secretion of HMGB1 through combination therapy. It’s worth noting that the NIR-irradiated CICF exhibited the lowest levels of green fluorescence of HMGB1, indicating an enhanced secretion of HMGB1 from CICF due to the targeting effect of FA (Fig. 5A and C). CRT represented another significant feature of ICD. The cells treated with CICF in combination with NIR exhibited a pronounced red fluorescence of CRT, surpassing that observed in cells treated with other groups. This suggests that CICF-mediated PTT and CDT can effectively induce an immunogenic response (Fig. 5B and D).

DCs are indispensable protagonists in anti-tumor immune response [42]. During ICD, signaling molecules such as CRT and HMGB1 are exposed on the surface of tumor cells, where they can be recognized by specific receptors on DCs, thereby facilitating the maturation of DCs [43]. To investigate the immunostimulatory effect of CICF on DCs maturation, we co-cultured mouse bone marrow-derived dendritic cells (BMDCs) with pretreated 4 T1 cells. The typical markers CD80 and CD86 were detected by flow cytometry (Fig. 5G, H). The CICF + NIR group elicited the most pronounced maturation of DCs, achieving a level 3.51 times greater than that observed in the control group. It is noteworthy that the DCs maturity in the CICF + NIR group is 1.52 times greater than that observed in the CICF group. This finding suggests that NIR can more effectively facilitate the release of tumor antigens in conjunction with CpG, thereby enhancing the efficacy of immunotherapy. Furthermore, the combination of CICF and NIR demonstrated a higher efficacy compared to CIC and NIR, providing additional evidence that CICF exhibits enhanced immunostimulatory activity following FA targeting. Mature DCs are capable of secreting immune-related cytokines, such as tumor necrosis factor-alpha (TNF-α) and interleukin-6 (IL-6). We subsequently assessed the secretion levels of TNF-α and IL-6 using enzyme-linked immunosorbent assays (ELISA) (Fig. 5E, F). The results indicated that the secretion levels of TNF-α and IL-6 induced by CICF were significantly elevated compared to those in cells treated with free CpG or CIC. This finding suggests that CIC and CICF effectively facilitate the intracellular delivery of CpG, thereby promoting the maturation of DCs and playing a crucial role in anti-tumor immunity.

### Targeting ability of CICF in vivo and photothermal imaging

**Fig. 6:**
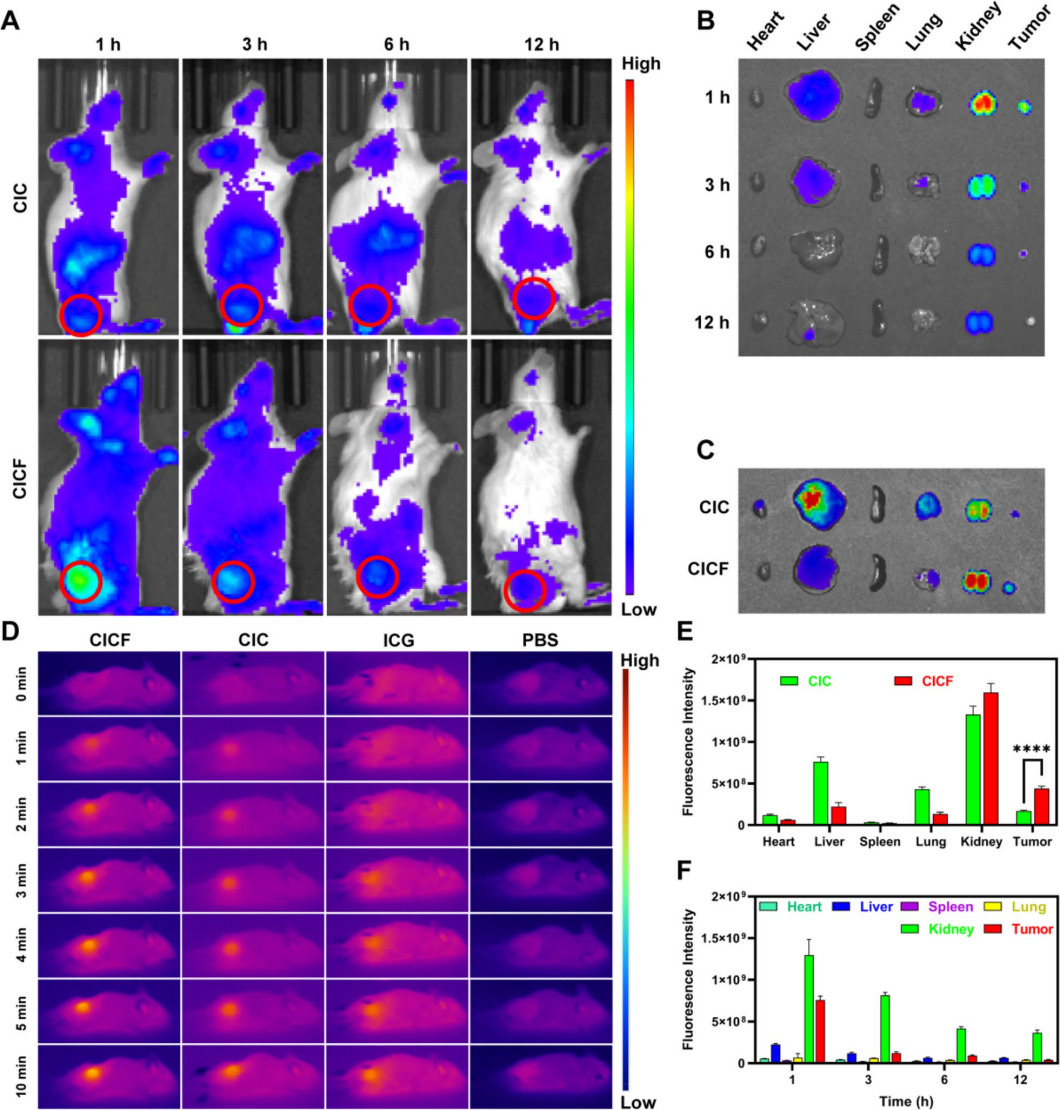
**A** The fluorescence distribution of 4 T1 tumor-bearing mice was observed after intravenous injection of CICF-Cy5 and CIC-Cy5 at 1, 3, 6, and 12 h. **B** Ex vivo fluorescence images of resected tumors and major organs at 1, 3, 6, and 12 h after CICF-Cy5 injection. **C** Ex vivo fluorescence images of tumors and major organs harvested 1 h after injection of CICF-Cy5 or CIC-Cy5. **D** Near-infrared thermal imaging of 4 T1 tumor-bearing mice at different time points after different treatments. **E** Semi-quantitative analysis of fluorescence intensity of Figure (**C**). **F** Semi-quantitative analysis of fluorescence intensity of Figure (**B**). Results are expressed as mean ± SD (n = 3). The difference was statistically significant: ****p < 0.0001

The favorable therapeutic outcomes in vitro motivated us to investigate the tumor accumulation capacity and photothermal properties of CICF in vivo. CICF (CpG was labeled with Cy5) was intravenously injected into the 4 T1 tumor-bearing mice with CIC as the control. The fluorescence intensity at the tumor site peaked at 1 h and then started to decline at 3 h (Figs. 6A and S14). Additionally, the fluorescence intensity of CICF was higher than that of CIC, demonstrating that the FA modification conferred better targeting ability. By euthanizing the mice, the major organs and tumor tissues were obtained for ex vivo imaging (Fig. 6B, C). The semi-quantitative findings from ex vivo imaging, as illustrated in Fig. 6E, F, were consistent with the whole-body fluorescence imaging shown in Fig. 6A. These results demonstrated that CICF achieved approximately a 2.2-fold enhancement in tumoral fluorescence intensity relative to CIC at 1 h post-injection, thereby indicating the effective tumor-targeted delivery capability of CICF. Similar results were reported for in vivo bioimaging evaluation of nanoparticles [44].

Given the satisfactory tumor-targeting capacity of CICF in vivo, we subsequently evaluated the photothermal effect of CICF on 4 T1 tumor-bearing mice. When the tumor volume reached 150–300 mm^3^, PBS, ICG, CIC, and CICF were intravenously injected into the mice. One hour after intravenous injection, the tumors were irradiated with an NIR laser, and the surface temperature of the tumors was monitored by infrared thermal imaging at 0, 1, 2, 3, 4, 5, and 10 to assess the PTT effect of each group (Figs. 6D and S15). Compared with the Control group, the tumor temperatures in the ICG group, CIC group, and CICF group significantly increased over time. Furthermore, the temperature observed in the CICF group exceeded that of the other groups, suggesting an enhanced potential for PTT in tumor eradication.

### In vivo therapeutic effects of CICF

**Fig. 7:**
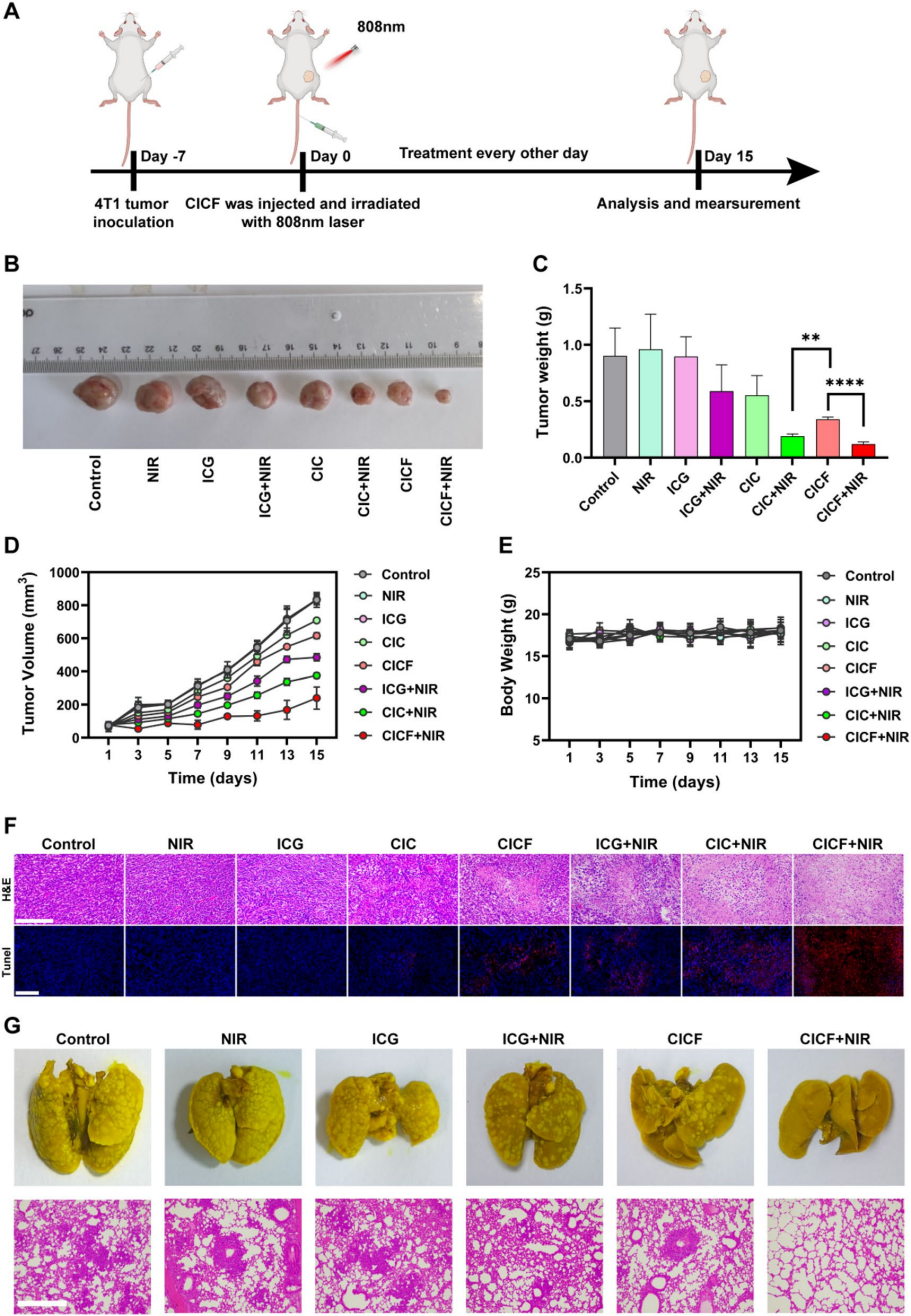
**A** Diagram of treatment in vivo in mice. **B** Representative photographs of removed tumors obtained on day 15 of mice in different treatment groups. **C** Average tumor weight after 15 days of treatment with different groups. **D** Tumor growth curves of 4 T1 tumor-bearing mice within 15 days after treatment with different groups. **E** Body weight changes of the mice during therapy. **F** H&E and TUNEL staining images of tumor sections of 4 T1 tumor-bearing mice. Scale bar: 200 μm. **G** Photographic and H&E staining images of the isolated lungs. Scale bar: 100 μm. Results are expressed as mean ± SD (n = 3). The difference was statistically significant: **p < 0.01, ****p < 0.0001

Encouraged by the efficient tumor accumulation capacity and excellent photothermal properties of CICF, we further evaluated the anti-tumor efficacy and in vivo safety of CICF in 4 T1 tumor-bearing mice (Fig. 7A). Mice were randomly divided into eight groups and treated with Control, NIR, ICG, ICG + NIR, CIC, CIC + NIR, CICF, and CICF + NIR, respectively. NIR laser irradiation was applied to mice at 1 h after tail vein injection. The changes in tumor volumes and body weights of the mice were monitored every 2 days for 15 consecutive days. The tumors in the Control, NIR, and ICG groups exhibited rapid growth, whereas tumor proliferation in the CIC and CICF groups was moderately suppressed. (Fig. 7B–D). This indicated that the laser irradiation and free ICG had no effect on tumor growth, and the CIC or CICF treatments had a poor therapeutic effect on tumors through CDT. The inhibitory effect in the CIC + NIR group was moderate, suggesting that the combined therapy of CDT and PTT had a better anti-tumor effect. The treatment regimen combining CICF with NIR demonstrated a significantly greater inhibition of tumor growth, suggesting that the introduction of tumor-targeting capabilities substantially enhanced antitumor efficacy. On the sixteenth day of the study, the mice were euthanized, and histopathological analyses were performed on their major organs, including tumor, heart, liver, spleen, lung, and kidney. The therapeutic efficacy of CICF was further assessed through hematoxylin and eosin (H&E) staining and terminal deoxynucleotidyl transferase-mediated dUTP nick end labeling (TUNEL) assay (Fig. 7F), which revealed significant nuclear necrosis or dissociation of tumor cells in the CICF + NIR group. Therefore, both H&E staining and TUNEL assay demonstrate that the CICF + NIR group exhibits significant anti-tumor efficacy. Additionally, to further assess the potential inhibition of lung metastasis, the lungs of 4 T1 tumor-bearing mice were harvested after 30 days of various treatments to evaluate treatment efficacy (Fig. 7G). No significant lung metastases were observed in the CICF + NIR-treated mice compared to other groups. Therefore, under near-infrared laser irradiation, CICF not only exerts chemodynamic therapy (CDT) effects but also induces photodynamic therapy (PDT) and photothermal therapy (PTT), thereby achieving a synergistic effect of CDT and PDT/PTT. This significantly enhances the efficiency of tumor growth inhibition and more effectively prevents lung metastasis. CICF system, combining CDT/PDT/PTT under NIR, aligns with multimodal nanotherapeutics (e.g., CuS nanoflakes, catalytic scaffolds) that disrupt tumor immunosuppression via synergistic ROS/hyperthermia-driven antigen release and immune activation. This nanoengineered strategy enhances tumor suppression and metastasis prevention, exemplifying the power of material-driven synergy in advancing precision cancer immunotherapy [29–31, 45].

### The antitumor immune response of CICF in vivo

**Fig. 8:**
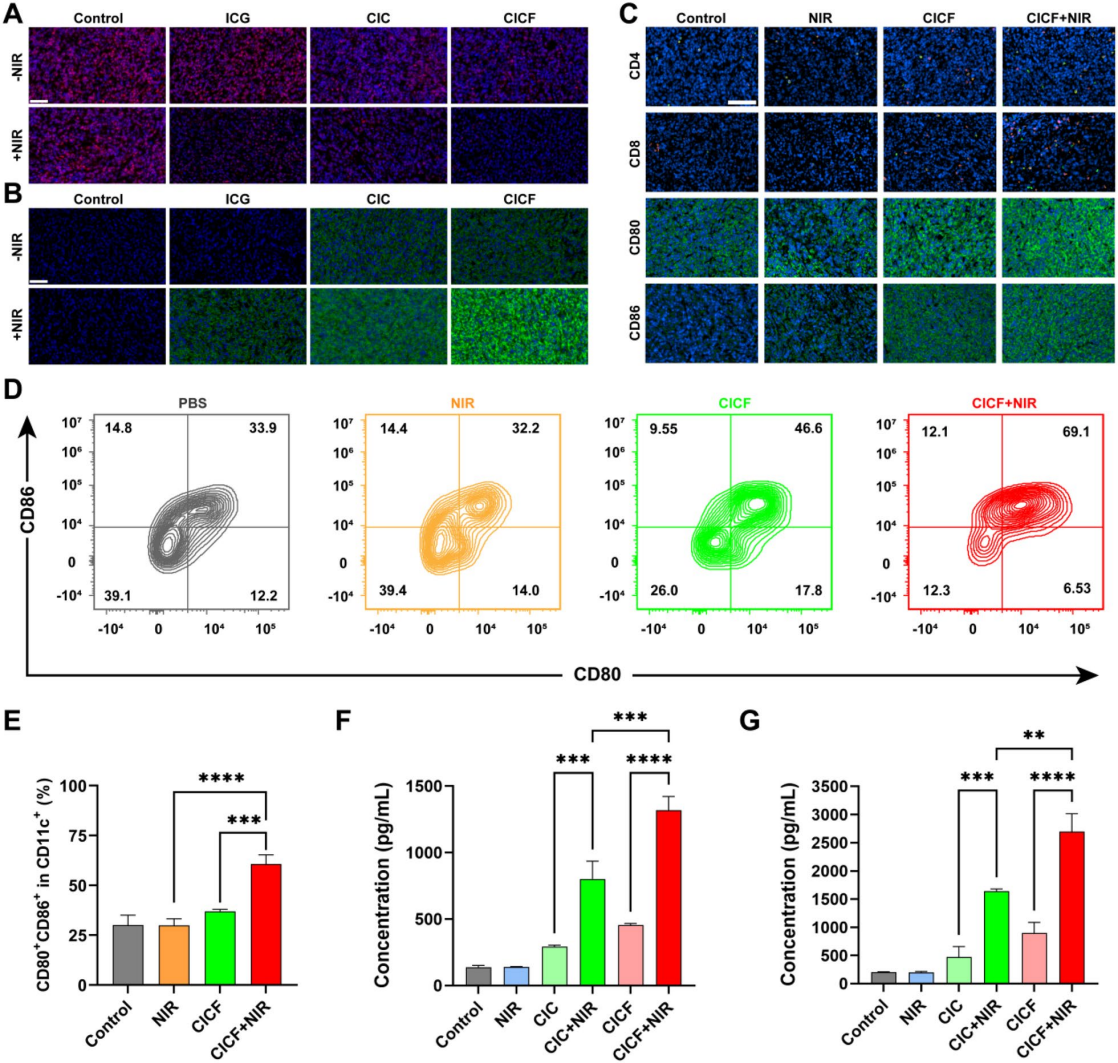
**A** HMGB1 and **B** CRT immunofluorescence staining images of tumor sections of 4 T1 tumor-bearing mice. Scale bar: 100 μm. **C** Immunofluorescence images of CD4 +, CD8 +, CD80 +, and CD86 + in tumor tissues. Scale bar: 100 μm. **D** The maturation of DCs in tumor tissues was detected by flow cytometry. **E** Quantification of the proportion of mature DCs within tumor tissue. Serum levels of **F** IL-6 and **G** TNF-α were detected after 15 days of treatment with different groups. Results are expressed as mean ± SD (n = 3). The difference was statistically significant: **p < 0.01, ***p < 0.001, ****p < 0.0001

Subsequently, we evaluated the antitumor immune response elicited by CICF through immunofluorescence staining of tumor sections. The tumor tissue treated with CICF + NIR showed a strong green fluorescence of CRT and low red fluorescence of HMGB1, which is consistent with the results at the cellular level (Fig. 8A). These results suggested that CICF possessed the capability to not only eradicate tumors but also effectively induce ICD in tumors. Subsequently, we analyzed the maturation of DCs in tumor tissues by flow cytometry (Fig. 8D, E). After NIR laser irradiation, the CICF + NIR group induced the highest level of DCs maturation thanks to the synergistic effect of CpG and ICG. The percentage of mature DCs (CD80 + CD86 + in CD11c +) increased from 37.2% to 76.8%. DCs maturation can further stimulate cytotoxic T lymphocytes (CTLs) and improve anti-tumor immune response. Next, we tried to determine the activation status of T cells in mice (Fig. 8C). The CICF + NIR group significantly increased the number of tumor-infiltrating CD4 + and CD8 + T cells compared to the Control group. In addition, the secretion of cytokines, such as IL-6 and TNF-α, is a key indicator of anti-tumor immune response. The levels of IL-6 and TNF-α in the serum of mice treated with CICF + NIR were significantly upregulated (Fig. 8F, G). The findings suggested that the NIR-irradiated CICF treatment was capable of eliciting a robust immunogenic response in vivo.

### Biosafety of CICF in vivo

The safety of CICF was further evaluated. All groups showed negligible changes in mouse weight for 15 days after treatments (Fig. 7E). Subsequently, to further assess the potential toxicity of CICF, we conducted histological examinations and hematological analyses. The results of H&E staining (Figure S16) revealed that there were no apparent pathological lesions in the vital organs of the treated mice compared to the control group, indicating that CICF treatment caused no significant damage to the organ structure of the mice. Furthermore, the serum biochemical indices (Figure S17) and complete blood cell counts (Figure S18) showed no significant differences between the treatment groups and the control group, further demonstrating that CICF not only possesses excellent anti-tumor efficacy but also exhibits good biocompatibility.

## Conclusion

Based on comprehensive research results, we have successfully developed a novel drug delivery nanosystem—CICF, by adopting self-assembly technology. The preparation of CICF entailed a one-step coordination-driven self-assembly process involving Cu^2^⁺ ions, CpG, and ICG, subsequently followed by surface modification with FA. The as-synthesized CICF realized the synergy of CDT and PTT/PDT through the integration of Cu^2+^ and ICG, thereby enhancing the therapeutic effect. Furthermore, CDT and PDT/PTT could induce ICD and therefore an antitumor immune response via apoptosis and necrosis. Both in vitro and in vivo results indicated that the CICF has outstanding biocompatibility, efficient tumor-targeting delivery capacity, and remarkable anti-tumor therapeutic benefits. In future research, we will primarily focus on the efficacy of Cu^2+^ clearance after injecting CICF into the body to determine the minimum dose that can effectively treat tumors without causing additional harm to the organism. Overall, we anticipate that this self-assembly construction strategy will promote the development of photo-induced antitumor synergistic therapy.

### Materials and methods

#### Materials

All oligonucleotides were synthesized and purified using HPLC by Sangon Biotechnology Co., Ltd (Shanghai, China). PEG-FA-COOH (17 kDa), indocyanine green (ICG), anhydrous copper chloride1 (CuCl_2_), and methylene blue (MB) were purchased from Sigma-Aldrich (St. Louis, MO, USA). Hoechst 33342 was obtained from Thermo Fisher Scientific (Waltham, MA, USA). The Cell Counting Kit-8 (CCK-8) and ROS assay kits were sourced from the Beyotime Institute of Biotechnology (Shanghai, China). The Annexin V-FITC/PI double-staining apoptosis detection kit was acquired from BestBio (Shanghai, China). ELISA kits were obtained from Solarbio Co., Ltd (Beijing, China). Fetal bovine serum (40130) was obtained from Yeasen Biotechnology (Shanghai) Co., Ltd. Other reagents were of analytical grade and were used without further purification.

#### Synthesis of CICF

First, 300 μL of a 25 μM CpG solution was prepared, and then 7.5 μL of a 25 mg/mL ICG solution was added to it, followed by vortexing for 10 s to ensure thorough mixing. Next, 30 μL of 10 mM anhydrous CuCl_2_ was added to the mixture, and it was vortexed again for 10 s. The resulting mixture was incubated in the dark at 65 °C for 1–2 h, ultimately assembling into CICs with a particle size of approximately 200 nm. Finally, to prepare CICF, 10 μL of a 2.5 mg/mL FA solution was added to the previously synthesized CIC, and the mixture was continuously stirred for 24 h to ensure thorough binding of FA to CIC. The CICF was washed several times by centrifugation, and the resulting precipitate was dispersed in deionized water and stored for further experiments. The synthesis steps for CIC were identical to those for CICF, except that FA was not included.

#### Characterization of the CICF

Transmission electron microscopy (TEM) images were obtained using a JEM-2100plus TEM microscope. Scanning Electron Microscopy (SEM) images were captured with a Hitachi S4800 SEM microscope. High-angle annular dark field scanning transmission electron microscopy (HAADF-STEM) images and elemental mapping were collected using a JEOL JEM-F200X TEM microscope. The zeta potential and size distribution of the CICF and CIC were analyzed using a Brookhaven 90Plus PALS. Ultraviolet–visible (UV–vis) absorbance was measured with an Edinburgh Instruments DS5 real-time dual beam UV/Vis spectrophotometer.

#### Determination of CpG and ICG loading efficiency

The supernatant was collected and the content of ICG in CICF was determined using UV–visible spectroscopy at 778 nm. The loading efficiency of ICG was calculated using the following formula: Loading efficiency = ((M0−MX)/M0) × 100%, where (M0) is the amount of ICG in the supernatant and (MX) is the total amount of ICG added during the initial phase of CICF synthesis. The ICG content in CIC was determined using the same method.

#### Evaluation of •OH, ^1^O_2_ production, and GSH consumption

The ability of CICF to generate •OH in vitro was assessed using an MB degradation assay. CICF, CIC, and Cu^2+^ (all containing the same amount of Cu^2+^, [Cu^2+^] = 2 μmol) were mixed with a GSH solution (0.5 mM, 500 μL) and incubated for 20 min at 37 °C. The mixture was then incubated with methylene blue (MB) (10 μg/mL, 1 mL) and H_2_O_2_ (10 mM, 500 μL) for an additional 30 min. After centrifugation, the absorbance of the supernatants was measured by UV–vis spectroscopy at 665 nm.

To detect ^1^O_2_, a solution of 20 μL of 10 mM diphenylisobenzofuran (DPBF) was added to 1 mL of the CICF sample (100 μg/mL). The sample was then irradiated with an 808 nm laser at a power density of 1.0 W/cm^2^ for 10 min. The absorption spectrum was obtained at specified time intervals using a UV–vis-NIR spectrophotometer.

The capacity of CICF to consume GSH was evaluated using DTNB. Under dark conditions, different concentrations of CICF were added to a solution of GSH (10 mM) and stirred vigorously. The mixture was then filtered, and the UV–vis spectrum of the supernatant was measured at 412 nm after adding DTNB (118 μg/mL).

#### Determination of photothermal efficiency

The photothermal performance of CICF was evaluated using a thermal infrared imager. Aqueous solutions of CICF at different concentrations (0, 25, and 50 μg/mL) were irradiated with an 808 nm laser (1.0 W/cm^2^), and the temperature of the solutions was measured using the thermal infrared imager. In addition, the photothermal properties of CIC and CICF were assessed at the same ICG concentration, and the photothermal conversion efficiency (η) of CICF was calculated. Finally, the photothermal stability of CICF was evaluated through a photothermal cycling experiment involving five cycles of laser switching. In each cycle, an aqueous solution of the material (50 μg/mL) was irradiated with the 808 nm laser for 5 min and then allowed to cool naturally to room temperature without the laser.

#### Measurement of cellular uptake of CICF

4 T1 cells were cultured in 1640 medium supplemented with 10% fetal bovine serum and 1% penicillin/streptomycin at 37 °C in a humidified atmosphere containing 5% carbon dioxide. Cell internalization of the 4 T1 cell line was detected using confocal microscopy and flow cytometry. The 4 T1 cells were seeded into confocal petri dishes or 6-well plates and incubated overnight. After incubation, the cells were treated with different drugs for 4 h. Following three washes with PBS, the samples were stained with Hoechst 33342 at room temperature (25 °C) for 10 min to label the nuclei and then imaged using a confocal laser scanning microscope. At the same time, the cells were trypsinized and suspended in 500 μL of PBS for flow cytometry analysis.

#### Detection of ROS production and cytotoxicity of CICF in vitro

The 4 T1 cells were counted using a Countstar automated cell counter (Countstar Mira FL, Shanghai, China) and seeded in 12-well plates and confocal dishes at a density of 2 × 10^4^ cells per well and cultured overnight. The 4 T1 cells were divided into eight groups: (1) Control; (2) NIR; (3) ICG; (4) CIC; (5) CICF; (6) ICG + NIR; (7) CIC + NIR; (8) CICF + NIR. After treatment, the cells were incubated with 1640 medium containing DCFH-DA for 30 min, followed by three washes with PBS to remove excess DCFH-DA. Finally, CLSM and flow cytometry were employed to detect DCFH-DA in the cells, enabling the observation of reactive oxygen species (ROS) production in living cells.

The cytotoxicity of the CICF was assessed using CCK-8 assays. 4 T1 cells were seeded at a density of 1 × 10^4^ cells per well in 96-well plates and incubated for 24 h. Then, the cells were treated with various concentrations of ICG, CIC, and CICF for 4 h. Following treatment, the cells were irradiated with an 808 nm laser (1 W/cm^2^) for 5 min and then incubated overnight. After incubation, the supernatant was discarded, and the cells were treated with the prepared CCK-8 reagent for 1 h. The absorbance was measured at a wavelength of 450 nm following the incubation.

4 T1 cells were seeded onto a 35 mm confocal dish at a density of 1.5 × 10^4^ cells per dish and allowed to incubate overnight to facilitate adherence to the substrate. Subsequently, various agents were introduced and incubated for 4 h. Following the incubation, the cells were washed three times with PBS to remove any residual drug. The cells were then subjected to co-staining with Calcein-AM and PI for 30 min, followed by additional washes with PBS. Fluorescence imaging was performed using CLSM.

#### Evaluation of HMGB1, CRT, and cytokines secretion

4 T1 cells were seeded in a confocal dish and allowed to incubate overnight. The cell culture medium was then replaced with PBS, and treated with ICG, CIC, and CICF. After 4 h incubation, the cells were irradiated with an 808 nm laser for 5 min and then further cultured for 12 h. The cell culture medium was removed, and the cells were washed three times with PBS. The cells were fixed with 4% paraformaldehyde (PFA) and permeabilized with 0.1% Triton X-100 for 10 min. Following three washes with PBS, the cells were incubated with 10% BSA for 30 min. The primary antibody against HMGB1 was applied for 1 h. After removing the antibodies, the cells were washed with PBS for 2 min and then rinsed three times. Subsequently, the cells were incubated with the secondary antibody for 1 h. After the removal of the secondary antibody, the cells were washed again with PBS for 2 min and underwent three additional washes. The cells were then stained with Hoechst 33342 staining solution for 10 min, followed by three more washes. Confocal images were acquired using a CLSM equipped with a 60 × objective lens, utilizing 405 nm (for nuclei, DAPI) and 488 nm (for HMGB1, Alexa Fluor 488) lasers. In a separate experiment, 4 T1 cells were also seeded in a confocal dish overnight, after which the cell culture medium was replaced with PBS and treated with ICG, CIC, and CICF. After another 4-h incubation and subsequent 5-min irradiation with an 808 nm laser, the cells underwent further culture for 12 h. The medium was removed, and cells were washed three times with PBS. They were then fixed with 4% PFA and permeabilized with 0.1% Triton X-100 for 10 min. After three washes, the cells were incubated overnight with an Alexa Fluor 647-conjugated anti-CRT antibody. Following this, the cells were washed three more times with PBS and stained with Hoechst 33342 for 10 min. After a final series of three washes, confocal images were acquired, using 405 nm and 647 nm lasers to visualize the nuclei (DAPI) and CRT (Alexa Fluor 647), respectively.

RAW264.7 macrophages were inoculated into 6-well plates at a density of 2 × 10^5^ cells per well. After incubating overnight, free CpG, CIC, and CICF were added to the cultures. Following a 24-h transfection period, the medium was collected and centrifuged at 4 °C at 1200 rpm for 15 min. Levels of IL-6 and TNF-α were analyzed using standard ELISA, following the manufacturer’s recommended protocol.

#### Evaluation of DCs maturation in vitro and in vivo

Bone marrow-derived dendritic cells (BMDCs) were isolated from BALB/c mice for this study. Initially, 4 T1 cells were seeded in a 6-well plate and incubated overnight. Subsequently, PBS and CICF were used in place of the standard culture medium. Following a 4-h incubation period, the tumor cells were irradiated using an 808 nm laser at an intensity of 1 W/cm^2^ for 5 min. The irradiated tumor cells were then harvested and co-cultured with BMDCs for 12 h. After the co-culture period, the BMDCs were collected, washed, and stained with monoclonal antibodies specific to CD11 C-FITC, CD80-PE, and CD86-PE-Cy5. Flow cytometry was then employed to analyze these cells.

To assess DCs maturation in vivo, mice were euthanized, and tumors were excised for analysis via flow cytometry. Tumor tissues were initially enzymatically dissociated into a single-cell suspension, followed by red blood cell lysis. The cell suspension was then incubated with Fc receptor-blocking antibodies to prevent non-specific binding. Finally, the cells were stained with antibodies against CD11c, CD80, and CD86 to facilitate flow cytometric analysis of DC maturation.

#### Biodistribution in vivo and photothermal imaging

All in vivo experiments were conducted in compliance with NIH guidelines, and the protocol was approved by the Institutional Animal Care and Use Committee of the Beijing Tuberculosis and Thoracic Tumor Research Institute. Female Balb/c mice aged 6–8 weeks (16–18 g) were purchased from Beijing Vital River Laboratory Animal Technology Co., Ltd. and maintained in a sterile environment. 4 T1 tumor-bearing BALB/c nude mice were intravenously injected with CIC-Cy5 or CICF-Cy5 for biodistribution studies. Whole-body imaging of the mice was performed using the IVIS imaging system at selected time points after injection (1, 3, 6, and 12 h). Twenty-four hours post-injection, tumor tissue, along with the principal organs (heart, liver, spleen, lungs, and kidneys), was excised from the euthanized mice and underwent ex vivo imaging analysis.

For in vivo photothermal imaging, tumor sites were irradiated with an 808 nm laser at an intensity of 1 W/cm^2^ for 5 min, administered 1 hour after the injection of PBS, ICG, CIC, or CICF. The resulting temperature variations within the tumor tissue were systematically monitored using a thermal camera.

#### Evaluation of the in vivo antitumor efficacy of CICF

Upon the tumor volume reaching approximately 50 mm^3^, the mice were randomly allocated into eight groups. Each group was administered a distinct intravenous treatment, which included PBS, ICG, CIC, or CICF, on a single occasion. Light irradiation was conducted 1 hour post-injection. The body weight and tumor volume of the mice were monitored bi-daily. Tumor volume was quantified using calipers and calculated according to the formula: V = (a*b^2^)/2, where ‘a’ represents the length and ‘b’ denotes the width. Fourteen days following the various treatments, the tumors were excised and weighed. Histological examinations of the tumors and major organs were performed using hematoxylin–eosin (H&E) and TUNEL assays.

#### Safety assessment of the CICF

After obtaining orbital venous blood from the mice, a Celltac MEK-6318 K was used to measure the red blood cell count (RBC), hematocrit (HCT), and hemoglobin concentration (MCHC). Whole blood was centrifuged at 3000 rpm for 10 min to obtain serum, which was used for ELISA assays and biochemical analysis.

#### Statistical analysis

The quantitative results of this study are shown as mean ± standard deviation (SD). Statistical analysis was performed using the GraphPad Prism software (version 8.0) using student t-tests or one-way analysis of variance (ANOVA). The significance was *p < 0.05, **p < 0.01, ***p < 0.001, ****p < 0.0001, respectively.

## Supplementary Information

Supplementary material 1

## Data Availability

No datasets were generated or analysed during the current study.
