# Supplementary material for: A minimalist self-assembly nanosystem for cancer immunotherapy via multiple immune activation
Source: J Nanobiotechnology. 2025 Jun 2;23:410. doi: 10.1186/s12951-025-03464-1 (PMC12131631; doi:10.1186/s12951-025-03464-1)
Supplement: Supplementary file 1 — Supplementary material 1 [file 12951_2025_3464_MOESM1_ESM.docx]

A minimalist self-assembly nanosystem for cancer immunotherapy via multiple immune activation

Weizhe Xu^a#^, Shiyuan Wang^b#^, Jiayi Zhang^c^, Fang Wang^c^, Zhaogang Sun^a,c^, Bei Liu^d^*, Jun Ye^b^*, Hongqian Chu^a,c^*

^a^Translational Medicine Center, Beijing Tuberculosis and Thoracic Tumor Research Institute, Beijing 101149, China;

^b^State Key Laboratory of Bioactive Substance and Function of Natural Medicines, Institute of Materia Medica, Chinese Academy of Medical Sciences & Peking Union Medical College, Beijing 100050, China;

^c^Translational Medicine Center, Beijing Chest Hospital, Capital Medical University, Beijing 101149, China;

^d^College of Science, Minzu University of China, Beijing 100081, China.

^#^Weizhe Xu and Shiyuan Wang contributed equally to this work and share the first authorship.

Correspondence: Professor Bei Liu (liubeinano@163.com), Jun Ye (yelinghao@imm.ac.cn), and Hongqian Chu (chuhongqian@bjxkyy.cn).


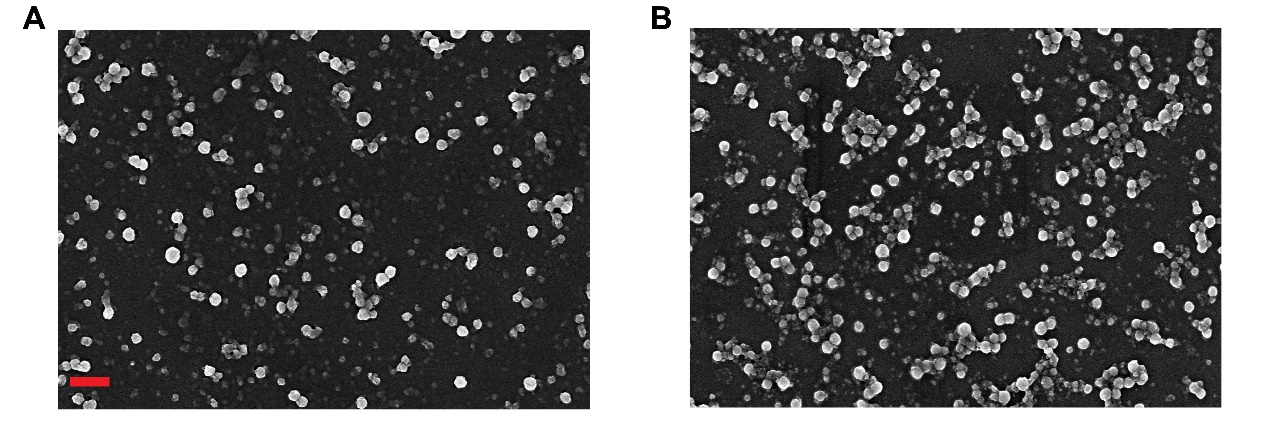


**Figure S1.** Morphologies of (A) CIC, and (B) CICF, revealed by scanning electron microscope (SEM). Scale bar: 500 nm.


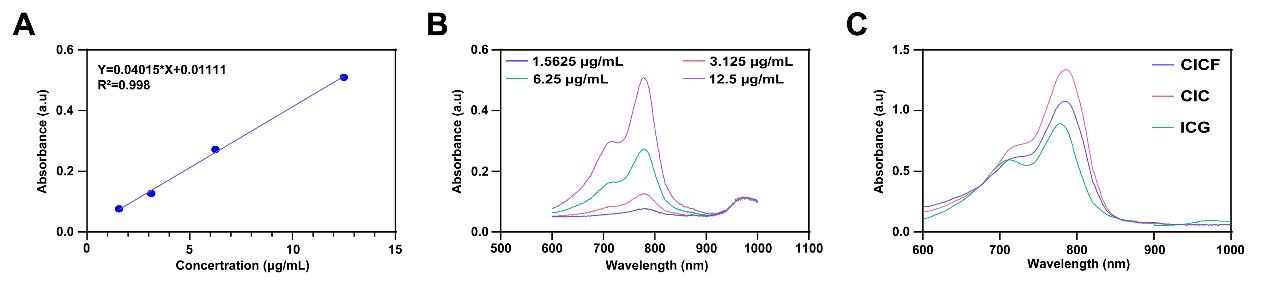


**Figure S2.** (A) The standard curve corresponding to ICG concentration. (B) UV–vis absorption spectra of ICG aqueous solutions with different concentrations. (C) UV-vis absorption spectra of ICG, CIC, and CICF in water.


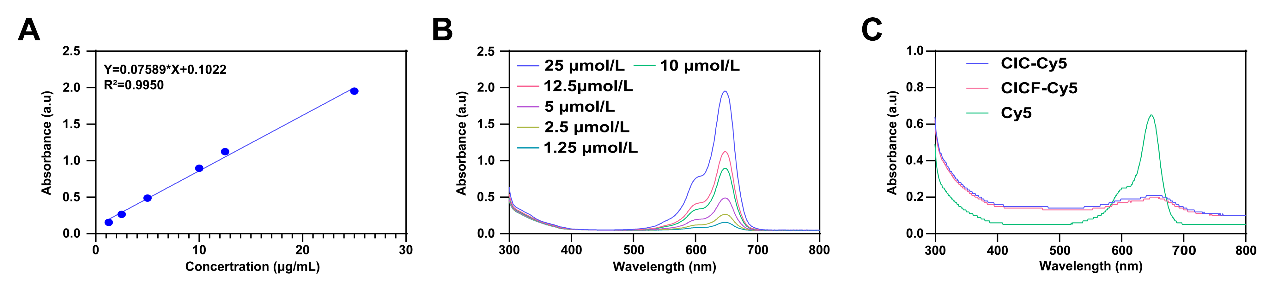


**Figure S3.** (A) The standard curve corresponding to Cy5-CpG concentration. (B) UV–vis absorption spectra of Cy5-CpG aqueous solutions with different concentrations. (C) UV-vis absorption spectra of Cy5-CpG, CIC-Cy5, and CICF-Cy5 in water.


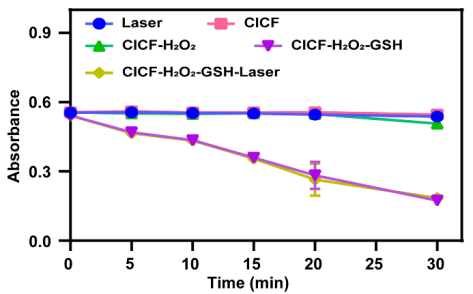


**Figure S4.** UV–vis light changes of MB aqueous solution within 30 min treated by different treatment methods and 10 mM GSH. Results are expressed as mean ± SD (n = 3).


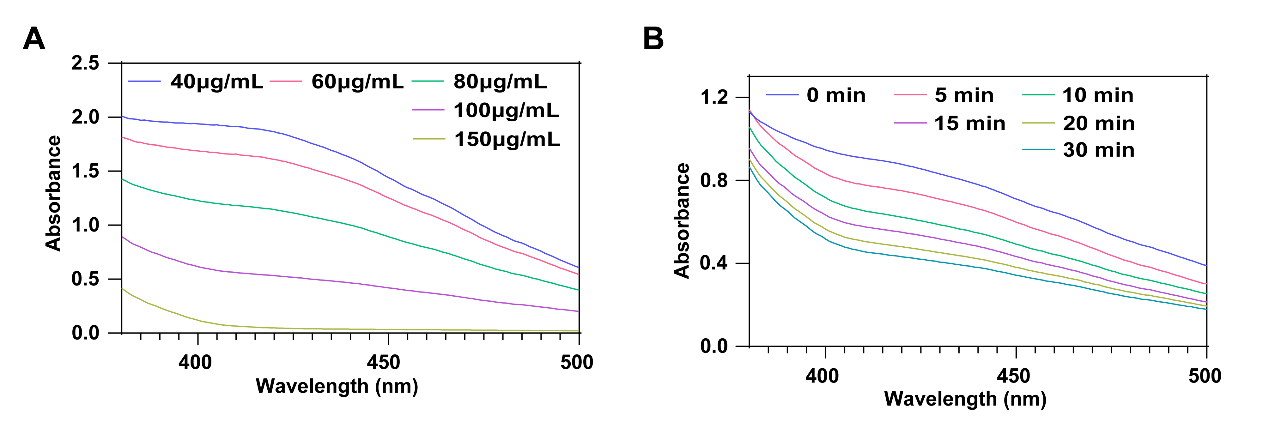


**Figure S5.** (A) UV–vis absorption spectra of GSH consumption with different concentrations of CICF NPs by the DTNB assay (B) UV–vis absorption spectra of GSH consumption within 30 min of 100 ug/ml CICF NPs by the DTNB assay.


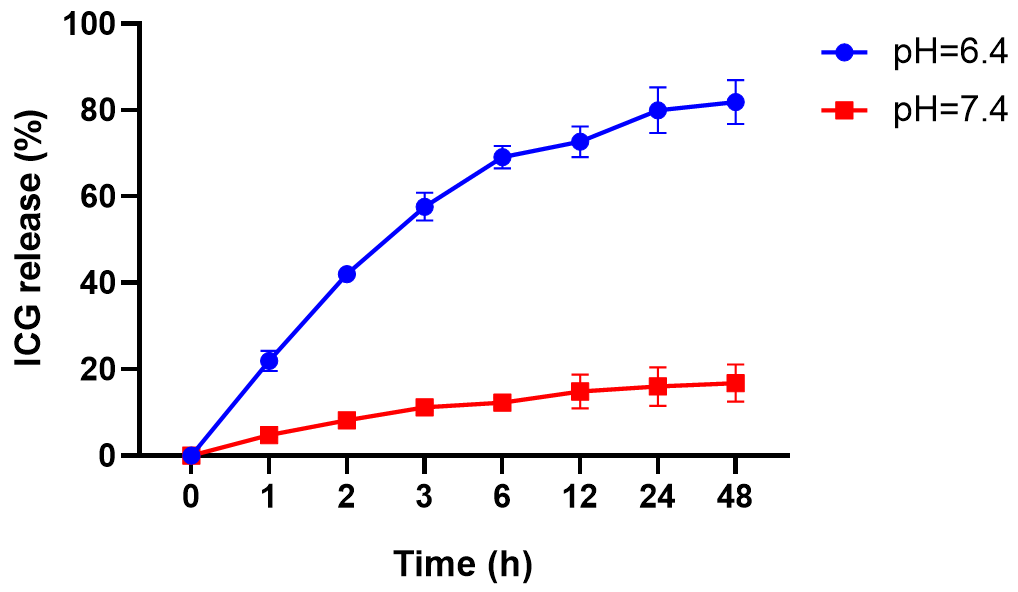
**Figure S6**. pH-dependent cumulative release profiles of ICG from CICF. Results are expressed as mean ± SD (n = 3).


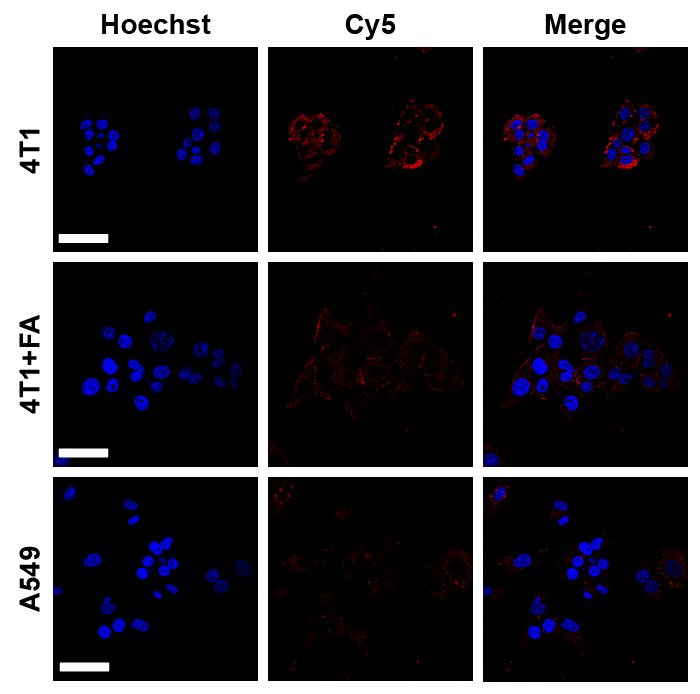


**Figure S7.** Confocal laser scanning microscopy (CLSM) analysis of Cy5-labeled CICF cellular uptake in different cell models: A549 cells (A549 group), 4T1 cells (4T1 group), and 4T1 cells with competitive FA inhibition (4T1+FA group). Scale bar: 50 μm.


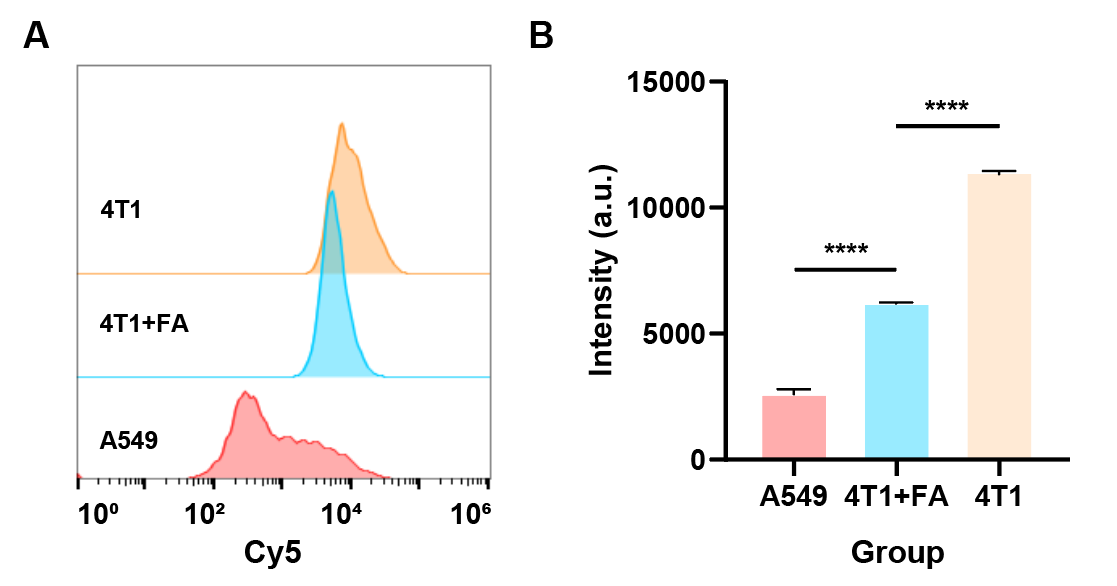


**Figure S8.** (A) Flow cytometric analysis of Cy5-labeled CICF cellular uptake in different cell models: A549 cells (A549 group), 4T1 cells (4T1 group), and 4T1 cells with competitive FA inhibition (4T1+FA group). (B) Corresponding normalized mean fluorescence intensity (MFI) for (A). Results are expressed as mean ± SD (n = 3). The difference was statistically significant: ****p < 0.0001.


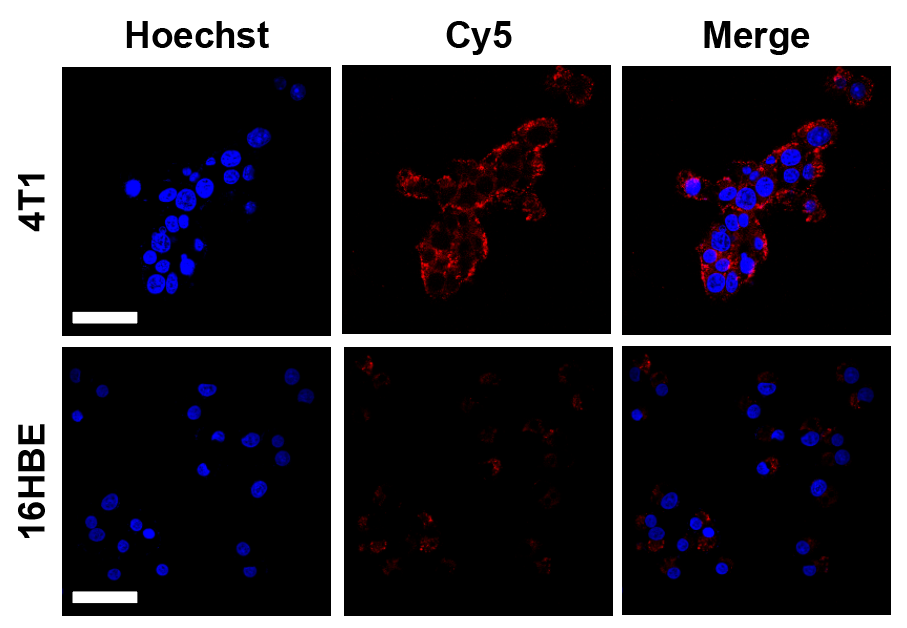


**Figure S9.** CLSM images of 16HBE cells and 4T1 cells incubated with Cy5-labeled CICF for 4 h. Scale bar: 50 μm.


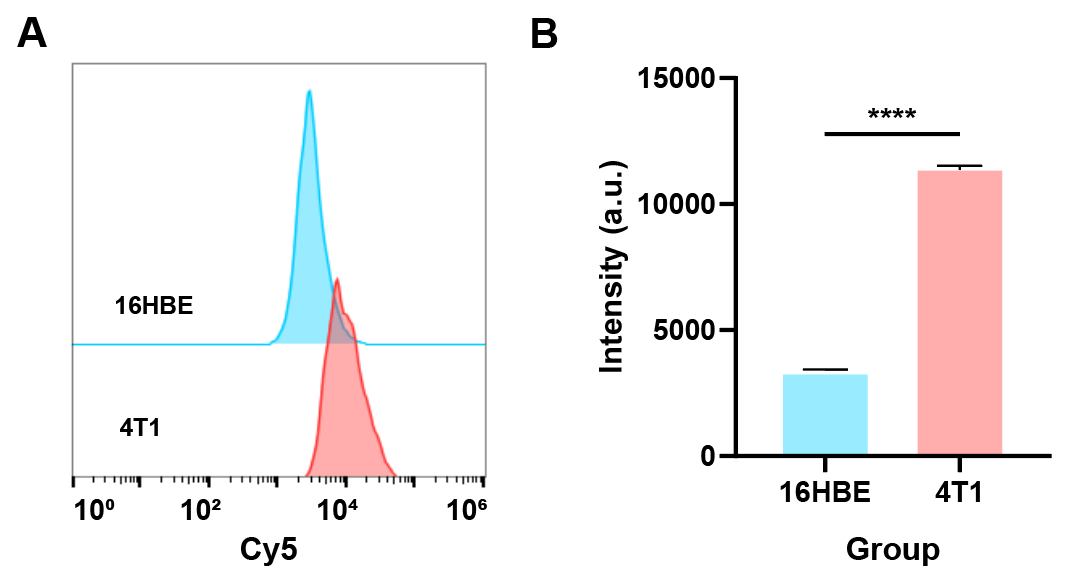


**Figure S10**. (A) Flow cytometry analysis of cellular uptake in 16HBE cells and 4T1 cells treated with Cy5-labeled CICF for 4 h. (B) Corresponding normalized mean fluorescence intensity (MFI) for (A). Results are expressed as mean ± SD (n = 3). The difference was statistically significant: ****p < 0.0001.


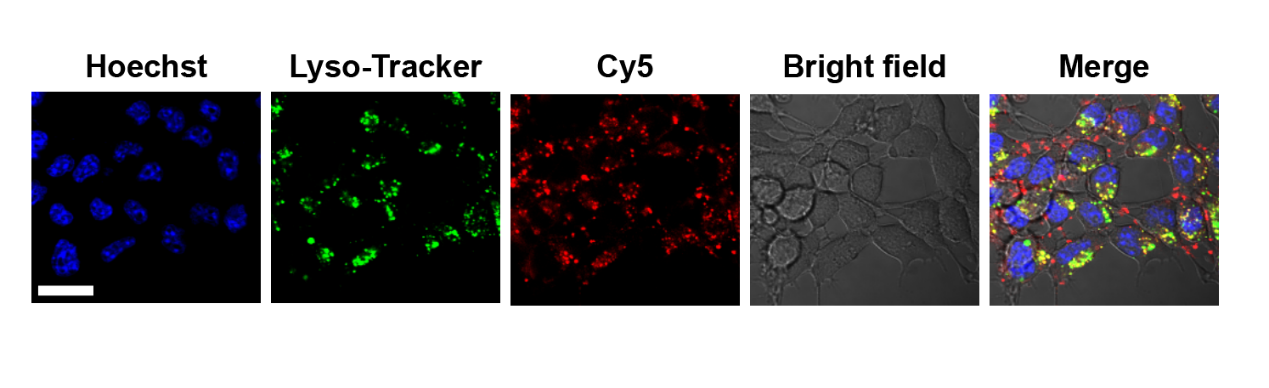
**Figure S11**. Subcellular localization of Cy5 labeled CICF. The lysosome was labeled with Lyso-Tracker green. Scale bar: 50 μm.


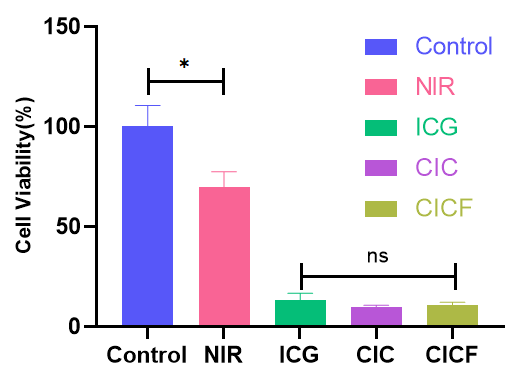


**Figure S12.** In vitro cytotoxicity of various drug treatments against 4T1 cells under NIR irradiation (2 W/cm²). Results are expressed as mean ± SD (n = 3). The difference was statistically significant: *p < 0.05.


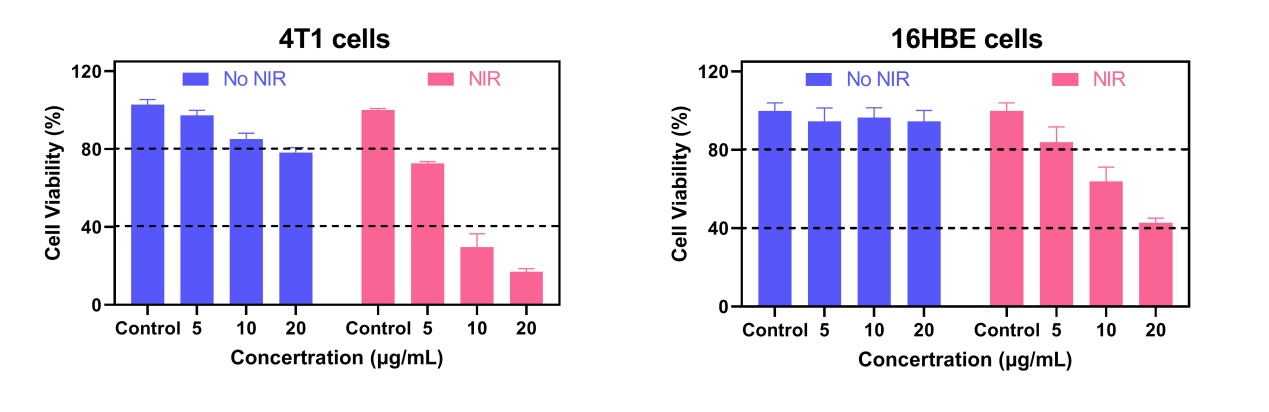


**Figure S13.** Cell viability of 4T1 (data from Figure 4B) and 16HBE cells treated with different concentrations of CICF (from 5 to 20 μg/mL) under laser or no laser conditions. Results are expressed as mean ± SD (n = 3).


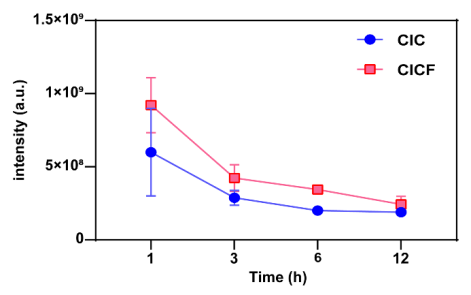


**Figure S14.** Fluorescence intensity quantification of tumor tissue after intravenous injection of CICF-Cy5 or CIC-Cy5 at different time points. Results are expressed as mean ± SD (n = 3).


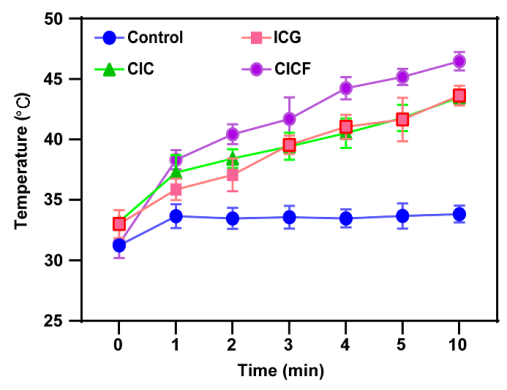


**Figure S15.** Temperature change curve of tumor site (6D). Results are expressed as mean ± SD (n = 3).


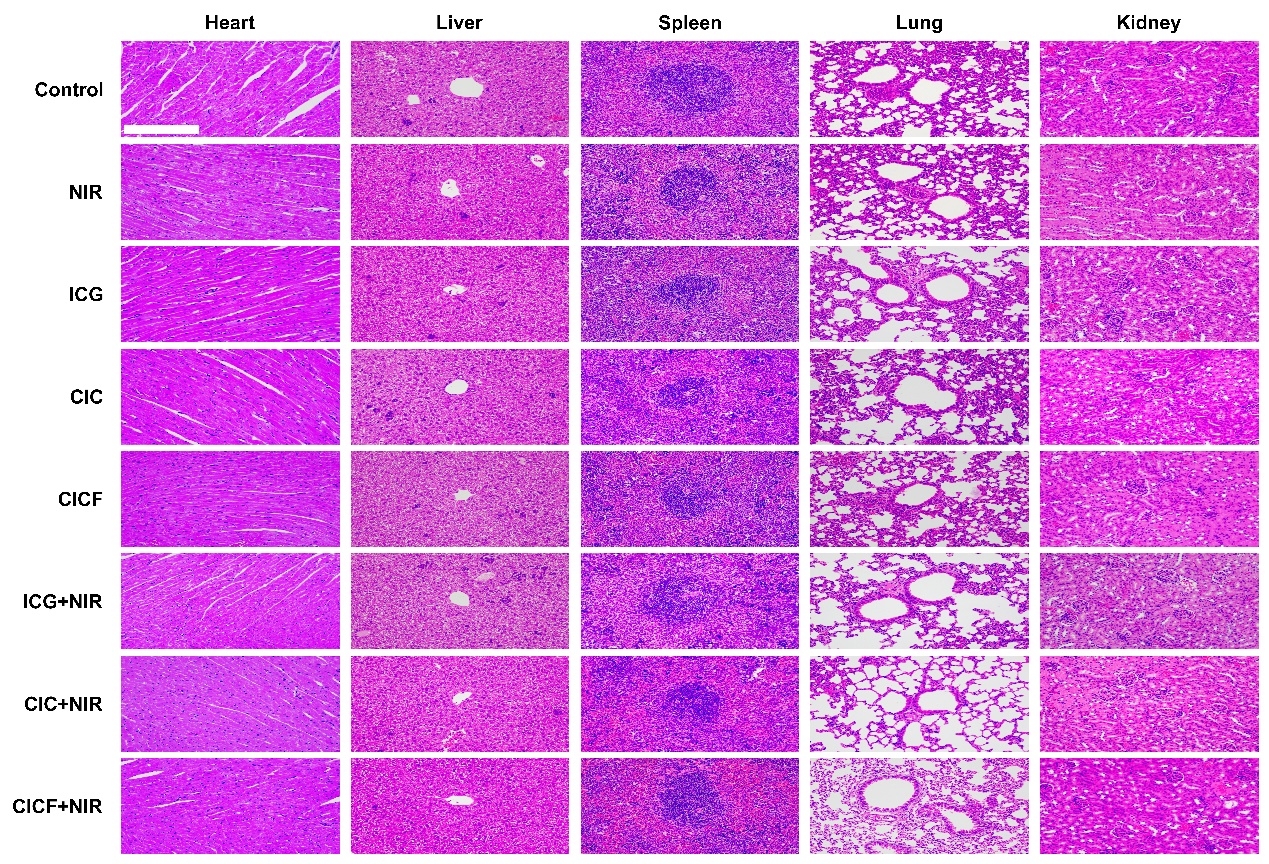


**Figure S16.** H&E staining image of 4T1 tumor-bearing mouse organ section. Scale bar: 200 μm.


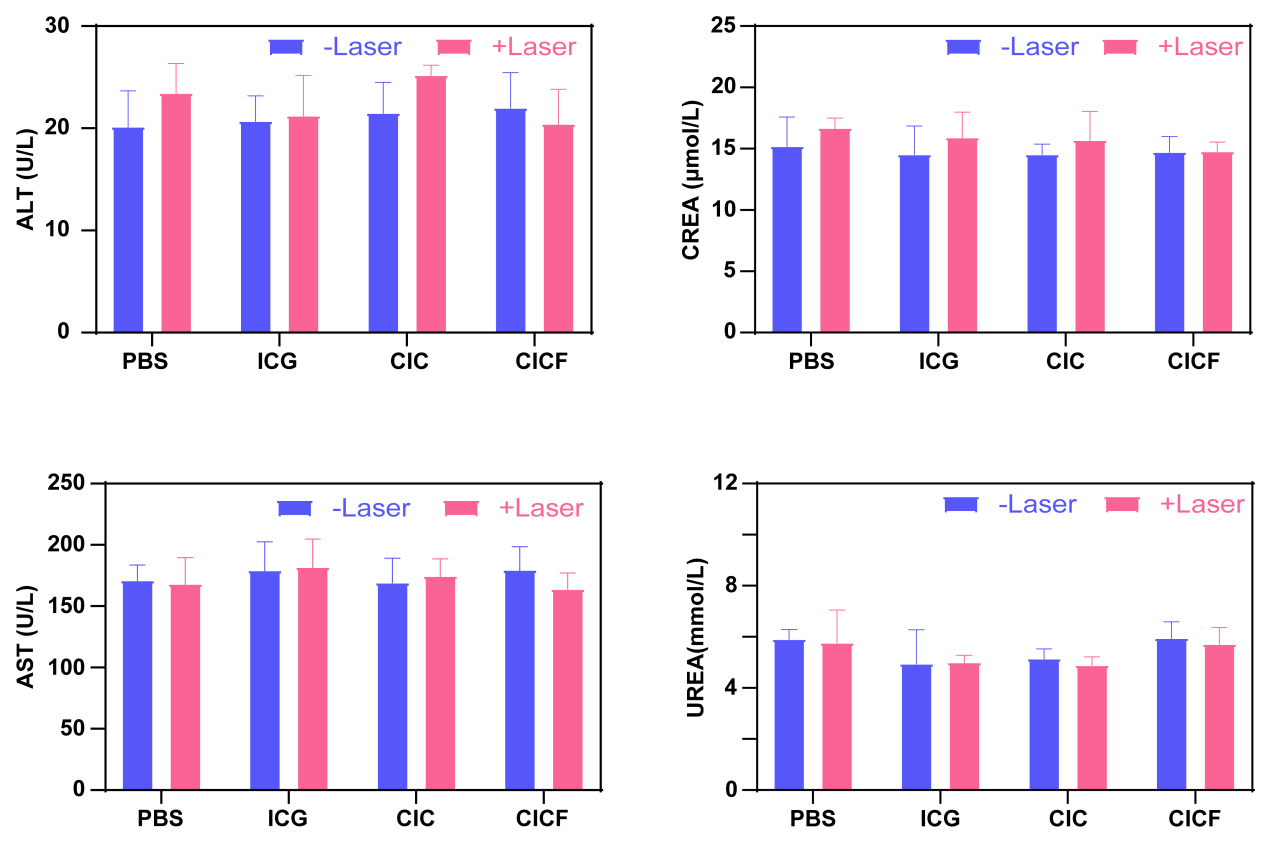


**Figure S17.** Blood biochemical indices of different groups. Results are expressed as mean ± SD (n = 3).


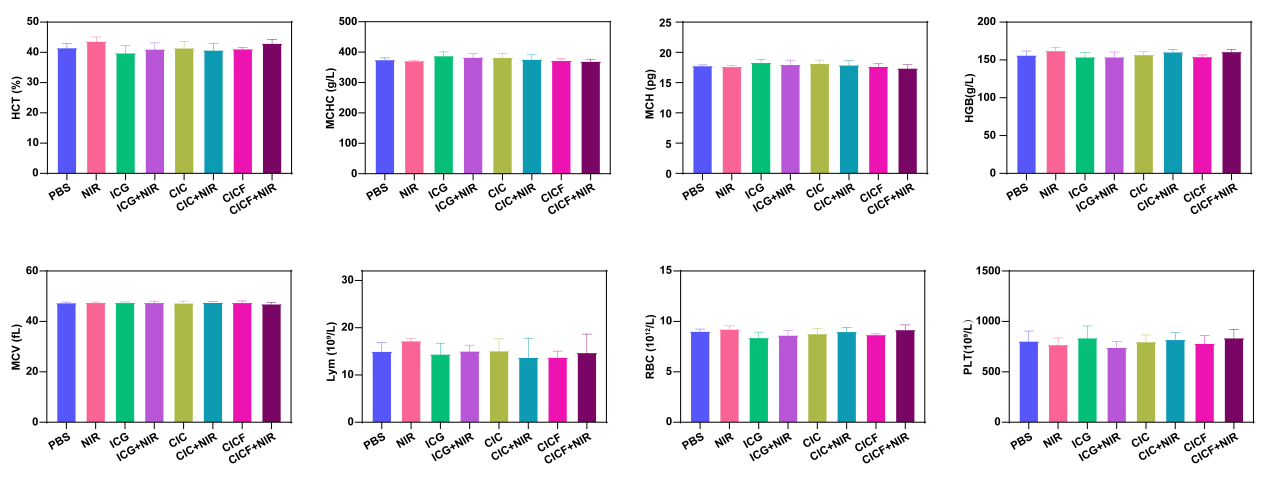


**Figure S18.** Blood routine indexes of different groups. Results are expressed as mean ± SD (n = 3).
